# Inhibition of SLC11A1‐Mediated Lysosomal Iron Accumulation in Microglia Promotes Repair Following White Matter Stroke

**DOI:** 10.1002/advs.202511482

**Published:** 2026-01-25

**Authors:** Lingling Qiu, Yajie Zhang, Yushi Tang, Hongli Hu, Ying Zhang, Junwen Xue, Hao Wang, Yecheng Wang, Chunfeng Liu, Jia Jia, Jian Cheng, Yongjun Cao

**Affiliations:** ^1^ Department of Neurology and Clinical Research Center of Neurological Disease The Second Affiliated Hospital of Soochow University Suzhou China; ^2^ Department of Neurology Taizhou Municipal Hospital (Taizhou University Affiliated Municipal Hospital) School of Medicine Taizhou University Taizhou China; ^3^ Department of Neurology Huai'an Fifth People's Hospital Huai'an Hospital Affiliated to Yangzhou University Huai'an China; ^4^ Jiangsu Key Laboratory of Drug Discovery and Translational Research For Brain Diseases Institute of Neuroscience Soochow University Suzhou China; ^5^ Jiangsu Key Laboratory of Drug Discovery and Translational Research For Brain Diseases College of Pharmaceutical Sciences Soochow University Suzhou China; ^6^ Suzhou International Joint Laboratory for Diagnosis and Treatment of Brain Diseases Soochow University Suzhou China

**Keywords:** iron accumulation, lysosomes, microglia, solute carrier family 11 member 1(SLC11A1), white matter stroke(WMS)

## Abstract

White matter stroke (WMS) results in demyelinating changes and neurological deficits. However, the underlying molecular mechanisms of demyelination after stroke and the specific role of microglia in white matter rehabilitation remain incompletely elucidated. This study identifies a time‐dependent accumulation of iron in microglial lysosomes mediated by solute carrier family 11 member 1 (SLC11A1), which persists from 12 h to 14 days following WMS. This iron accumulation results in damaged lysosomal myelin debris uptake and degradation in microglia. Notably, iron chelation with deferoxamine (DFO), microglia‐specific knockdown of SLC11A1, and administration of LM22B‐10, a SLC11A1 antagonist identified in this study, effectively reduce lysosomal iron accumulation in microglia, enhance microglial uptake and clearance of myelin debris, and ultimately promote functional recovery after WMS. Furthermore, SLC11A1 functions as a H^+^/Fe^2+^ antiporter that transports Fe^2+^ from the cytoplasm into lysosomes both in vitro and in vivo. Collectively, these results highlight that targeting SLC11A1 represents a previously unrecognized therapeutic strategy for WMS repair with significant clinical implications.

## Graphical abstract

Genetic and pharmacological inhibition of SLC11A1 functioning as an H^+^/Fe^2+^ antiporter–mediated lysosomal iron accumulation in microglia promotes lysosomal lumen acidification, increases CTSD expression, enhances lysosomal myelin debris uptake and degradation, and promotes repair following white matter stroke.

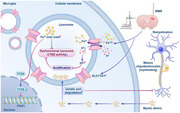

## 1 Introduction

White matter stroke (WMS), a common cerebrovascular condition, is responsible for approximately 25% of acute strokes, and it represents the second leading cause of dementia [1, 2]. To date, no effective therapy that promotes recovery following WMS has been identified [3]. A potential therapeutic strategy involves promoting the regeneration of oligodendrocytes (OLs) lost following WMS, specifically through inducing remyelination. Remyelination can directly address the core pathological issue of demyelination without inducing drug toxicity or affecting blood–brain barrier permeability, thereby improving neurological function. Although remyelination represents an endogenous mechanism for white matter damage repair, it requires the activation, proliferation, and differentiation of myelin‐producing cells. Oligodendrocyte progenitor cells (OPCs), the principal contributors to remyelination [4, 5, 6, 7], are widely distributed throughout the central nervous system (CNS). Their differentiation into mature OLs is essential for axon remyelination after demyelination. However, this differentiation, and the resulting remyelination, is a highly complex process that depends on a supportive and healthy microenvironment. After WMS, substantial myelin debris accumulation occurs in the affected area. Microglia, the resident immune cells of the CNS, can phagocytize cellular debris and remove myelin fragments [8], thereby maintaining an environment favorable for remyelination.

Solute carrier family 11 member 1 (SLC11A1), formerly known as natural resistance‐associated macrophage protein 1 (NRAMP1), is a divalent metal ion transporter primarily expressed on the lysosomal membrane of CNS microglia, in which it regulates iron homeostasis between lysosomes and the cytoplasm [9, 10]. Lysosomes are the principal organelles responsible for the uptake, digestion, and degradation of myelin debris by microglia during WMS repair [11]. However, lysosomes are also reservoirs of labile iron; excessive Fe^2+^ accumulation in microglial lysosomes promotes lipid peroxide formation, leading to lysosomal dysfunction [12]. As a bidirectional transporter of Fe^2+^, SLC11A1 mediates iron exchange across the lysosomal membrane. In the context of liver ischemia, SLC11A1 expression on the lysosomal membrane is increased, whereas its disruption ameliorates liver ischemia–reperfusion injury [13]. In WMS models, the directionality of Fe^2+^ transport by SLC11A1 remains undefined.

Previous studies have suggested that SLC11A1 transports Fe^2+^ from the cytoplasm into lysosomes while simultaneously exporting H^+^ from lysosomes into the cytoplasm. This activity disrupts lysosomal acidity and impairs the function of hydrolases essential for phagocytic and degradative processes [14]. Therefore, it has been hypothesized that, after WMS, SLC11A1 imports Fe^2+^ into lysosomes and exports H^+^, resulting in lysosomal dysfunction. The present study investigated the underlying mechanisms of WMS recovery from the perspective of microglial iron metabolism and transport and examined the role of SLC11A1 inhibition in facilitating recovery. The findings might provide a novel therapeutic target for small‑molecule inhibitors and gene therapies aiming to prevent and treat ischemic white matter injury.

## 2 Results

### 2.1 The Lysosomal Pathway and SLC11A1 Are Involved in the Pathophysiological Process of WMS

Injection of the vasoconstrictor N5‐(1‐iminoethyl)‐L‐ornithine (L‐NIO) into the corpus callosum induces white matter stroke (WMS). To dissect the molecular events in white matter lesions following WMS, we analyzed our bulk RNA‐sequencing (RNA‐seq) dataset generated from L‐NIO‐induced white matter lesions on day 7 post‐stroke, in comparison with control corpus callosum tissue from sham‐operated mice (GSE312001). Gene set enrichment analysis (GSEA) revealed activation of the lysosomal pathway (normalized enrichment score = 1.919, *p* < 0.005, *q* < 0.005), and the volcano plot indicated substantial upregulation of SLC11A1 expression in the L‐NIO group (Figure 2A; Figure S1B,C). To validate these bioinformatic findings and characterize the dynamic expression pattern of SLC11A1 at various time points post‐stroke, immunofluorescence (IF) staining was performed. As anticipated, the mean fluorescence intensity (MFI) of SLC11A1 in microglia gradually increased over time, peaking on day 7 post‐stroke and declining thereafter (Figure S1D,E). Consistent with these findings, western blotting identified a progressive increase in SLC11A1 protein expression in the ipsilateral corpus callosum from 12 h to 7 days post‐stroke, followed by a gradual decrease (Figure S1F,G). To identify key molecular determinants and potential mechanisms that differentially govern reparative versus non‐reparative outcomes in white matter injury, we injected L‐NIO into the corpus callosum to induce a non‐reparable WMS lesion, whereas lysophosphatidylcholine (LPC) was injected into the corpus callosum to induce reparable demyelination, which is consistent with previous findings [2, 15] (Figure S1A). Given that SLC11A1 predominantly localizes to lysosomal membranes in microglia within the CNS [9], we further performed western blotting and IF staining to compare lysosomal SLC11A1 expression between the L‐NIO and LPC models. The results demonstrated significantly higher SLC11A1 expression in irreparable L‐NIO lesions than in repairable LPC‐induced lesions (Figure 2B,C; Figure S1H,I). These findings indicated that the lysosomal pathway and SLC11A1 are critically involved in the pathophysiological process of WMS. Accordingly, we next examined the distinct mechanisms and pathological features of irreparable and repairable white matter injury before investigating specific roles of SLC11A1 in this context.

### 2.2 Iron and Myelin Debris Are Deposited in Activated Microglial Lysosomes within White Matter Lesions

In the context of WMS, cholesterol‐rich myelin debris extensively accumulates in the infarct core [8, 16]. IF staining revealed substantial myelin debris deposition within the lysosomes of activated microglia in the L‐NIO group at week 3 post‐stroke, whereas minimal myelin accumulation was observed in the LPC group (Figure S2A,B). Timely and effective clearance of myelin debris from demyelinated lesions by activated microglia is essential for the transition from a pro‐inflammatory environment to an anti‐inflammatory one, which facilitates the differentiation of OPCs into mature OLs [17, 18]. Conversely, extensive myelin debris remained in L‐NIO lesions at week 9 (Figure S2C,D), and neither pathological nor histological recovery was achieved (Figures S1A and S2E). IF staining illustrated that Caspr expression in ipsilateral lesions in the LPC group recovered to levels comparable to those on the contralateral side. However, in the L‐NIO group, Caspr expression remained considerably reduced, with little recovery observed by week 9 (Figure S2E). As SLC11A1 is associated with iron transport, we next investigated whether white matter injury is linked to iron accumulation within lysosomes. Previous analyses have implicated iron accumulation in ischemic stroke associated with middle cerebral artery occlusion [2, 19], yet its role in WMS remains poorly understood. Iron homeostasis is regulated by ferritin heavy chain (FTH) and ferritin light chain (FTL) for iron storage, transferrin receptor 1 (TFR1) for iron uptake, and ferroportin (FPN) for iron export [20]. Our findings demonstrated that the expression of iron metabolism‐related proteins (TFR1, FPN, FTH, and FTL), acyl‐CoA synthetase long‐chain family member 4 (ACSL4), and the glutathione system component glutathione peroxidase 4 (GPX4) and levels of lipid peroxidation products such as 4‐hydroxynonenal (4‐HNE) varied over time after WMS (Figure S2F–M). Semiquantitative analysis revealed that FTH and FTL expression progressively increased after stroke, peaking on day 7 (Figure S2F–H), mirroring the expression pattern of SLC11A1 (Figure S1F,G) and aligning with findings of iron accumulation in spinal cord white matter lesions [21].

We subsequently compared iron metabolism, lipid peroxidation, and glutathione‐related pathways between the irreparable L‐NIO and repairable LPC models. IF analysis revealed iron accumulation within the lysosomes of activated microglia in the L‐NIO group, but not in the LPC group, on day 7 post‐stroke (Figure S3A–F, the tissues were collected and analyzed on day 7 post‐stroke). Western blotting revealed increased FTH, FTL, TFR1, 4‐HNE, FPN, and ACSL4 expression, along with decreased GPX4 expression, in the L‐NIO group post‐stroke (Figure S3G–N, the tissues were collected and analyzed for FTH, FTL, and 4‐HNE on day 7 post‐stroke; for ACSL4, TFR1, and GPX4 on day 4; for FPN on day 2). However, no significant alterations were observed in the LPC group (Figure S3A–N).

### 2.3 Iron Chelation Enhances Lysosomal Uptake and Degradation of Myelin Debris, Promoting Injured White Matter Recovery

**FIGURE 1:**
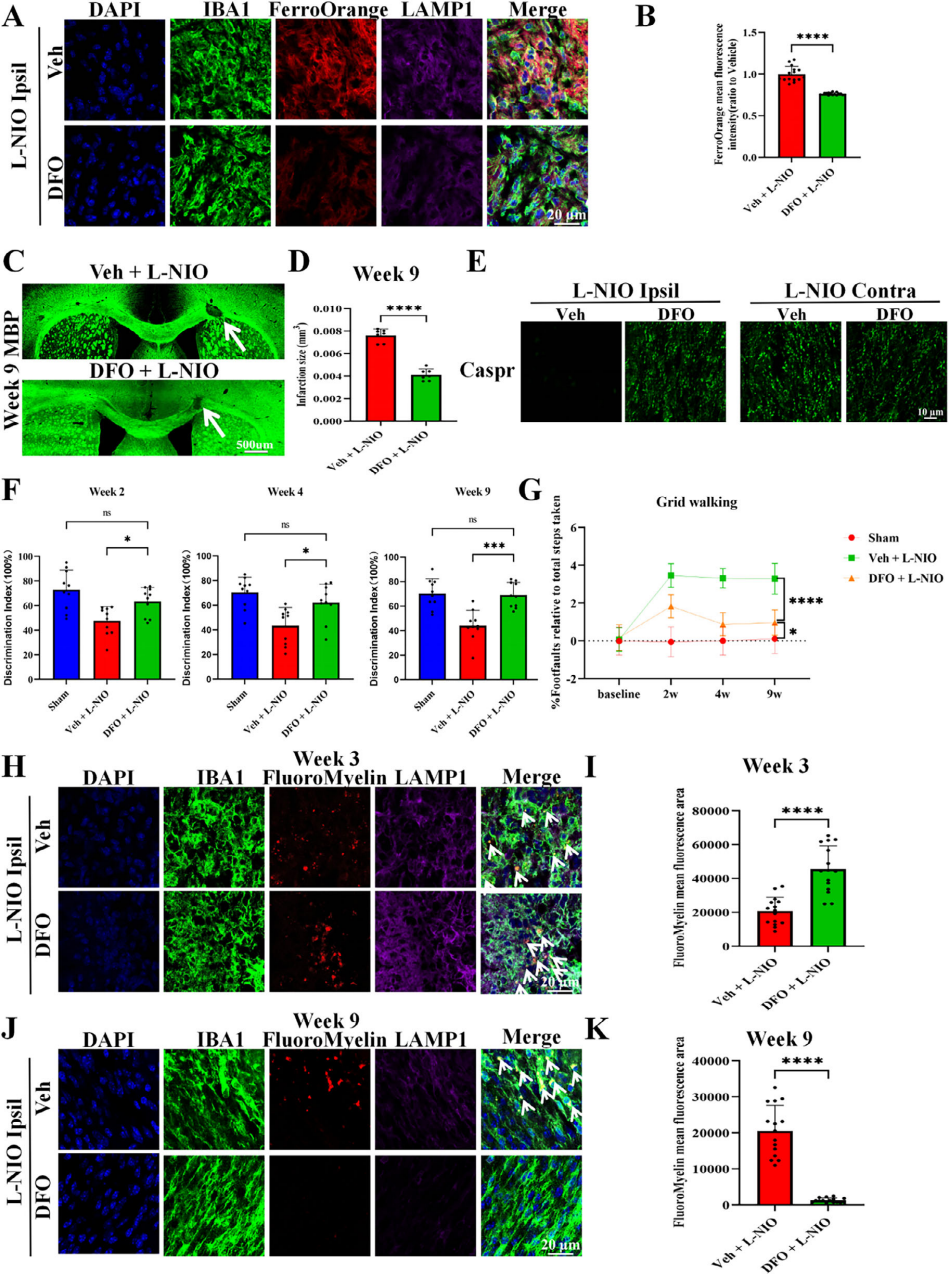
Iron chelation enhances lysosomal myelin debris uptake and degradation, supporting white matter recovery. (A,B) Representative images of DAPI (blue), IBA1 (green), FerroOrange (red, Fe^2+^), and LAMP1 (purple) staining on day 7 post‐stroke (A; scale bar = 20 µm) in the DFO and control groups and the corresponding quantification (B; each dot represents the average of three fields per brain section, *n* = 3 brain sections per mouse, N = 5 mice per group). Statistical analysis was performed using the two‐tailed unpaired Student's *t*‐test with Welch's correction. 95% *CI*[‐0.2868, ‐0.1852]. (C,D) IF staining revealing infarct lesions at week 9 post‐stroke in the DFO and control groups (C: white arrows indicate lesions; scale bar = 500 µm). and the corresponding quantification (D; N = 7; two‐tailed unpaired Student's *t*‐test; 95% *CI*[‐0.004134, ‐0.002882]). (E) Representative images revealing Caspr expression in various groups (three fields per brain section, *n* = 3 brain sections per mouse, N = 5 mice per group; scale bar = 10 µm). (F,G) Novel object recognition (F) and grid walking tests (G) at weeks 2, 4, and 9 post‐stroke (N = 10; one‐way ANOVA followed by Tukey's post hoc test; F: Veh+L‐NIO vs DFO+L‐NIO: Week 2 95% *CI*[‐30.43, ‐1.082], Week 4 95% *CI*[‐34.39, ‐3.195], Week 9 95% *CI*[‐37.81, ‐12.16]; G: Veh+L‐NIO vs DFO+L‐NIO: Week 9 95% *CI*[0.6620, 2.694]). (H,I) Representative images of DAPI (blue), IBA1 (green), FluoroMyelin (red), and LAMP1 (purple) staining at week 3 post‐stroke (H; scale bar = 20 µm) and the corresponding quantification (I; each dot represents the average of three fields per brain section, *n* = 3 brain sections per mouse, N = 5 mice per group; two‐tailed unpaired Student's *t*‐test; 95% *CI*[16375, 33318]). (J,K) Representative images of DAPI (blue), IBA1 (green), FluoroMyelin (red), and LAMP1 (purple) staining at week 9 post‐stroke (J; scale bar = 20 µm) and the corresponding quantification (K; each dot represents the average of three fields per brain section, *n* = 3 brain sections per mouse, N = 5 mice per group; two‐tailed unpaired Student's *t*‐test with Welch's correction; 95% *CI*[‐23235, ‐15378]).White arrows indicate colocalization of FluoroMyelin with LAMP1 and IBA1. Veh, vehicle. Data are presented as the mean ± SEM. *p < 0.05; ***p < 0.001; ****p < 0.0001; ns, not significant.

Given that iron accumulation in the lysosomes of activated microglia was associated with non‐reparable white matter lesions following WMS, we hypothesized that iron overload constitutes a key mechanism underlying the failure of remyelination in the WMS model. We therefore investigated whether the iron chelator deferoxamine (DFO) could promote white matter recovery in the L‐NIO model. According to prior studies, DFO alleviates intracellular and lysosomal iron accumulation, regulates iron metabolism‐related proteins, and reduces reactive oxygen species (ROS) generation following iron overload [22, 23, 24, 25, 26, 27]. IF analysis demonstrated that DFO administration reduced the MFI of FerroOrange (a marker for Fe^2+^), FTH, and FTL, indicating that DFO attenuated L‐NIO–induced iron accumulation in the lysosomes of activated microglia (Figure 1A,B; Figure S4A–D). Consistent with these findings, western blotting revealed decreased levels of iron metabolism‐related proteins (FTH, FTL, TFR1) and ROS‐associated markers (4‐HNE, ACSL4), along with increased levels of FPN and GPX4, in the DFO group relative to the vehicle group (Figure S4E–L). Given the observed reduction in iron accumulation after DFO treatment, we next explored whether DFO enhanced the lysosomal uptake and degradation of myelin debris in microglia. The results demonstrated that DFO treatment significantly reduced infarct volumes in the corpus callosum, and both histological and neurological recovery were substantially better in the DFO group than in the vehicle group (Figure 1C–G). Previous studies [16, 28] and our data suggest that removal of lipid‐rich myelin debris is essential for remyelination and white matter recovery. Therefore, we used ionized calcium‐binding adaptor molecule 1 (IBA1), lysosomal‐associated membrane protein 1 (LAMP1), and FluoroMyelin to label microglia, lysosomes, and myelin debris, respectively. As demonstrated by confocal microscopy, activated microglia in the DFO‐treated group exhibited greater engulfment of myelin debris at week 3 (Figure 1H,I) and enhanced degradation capacity relative to that of the controls (Figure 1J,K). At week 9 post‐stroke, myelin debris was abundantly deposited in microglia in the vehicle group, whereas near‐complete clearance was observed in the DFO‐treated group (Figure 1J,K).

These data provide strong evidence that the reduction of iron accumulation within the lysosomes of activated microglia is closely associated with their capacity to engulf and degrade myelin debris, which is essential for subsequent remyelination and white matter recovery after WMS.

### 2.4 *Slc11a1* knockdown Promotes White Matter Repair Following WMS

SLC11A1 plays a time‐dependent role in the pathophysiology of WMS (Figure S1D–G) and participates in iron transport between lysosomes and the cytoplasm [14]. Based on these findings, we hypothesized that *Slc11a1* knockdown would promote white matter recovery. To test this, we stereotactically injected either pCLenti‐U6‐*Slc11a1*‐short hairpin (shRNA)‐cytomegalovirus (CMV)‐enhanced green fluorescent protein (eGFP)‐woodchuck hepatitis virus post‐transcriptional regulatory element (WPRE) vectors or pCLenti‐U6‐negative control (NC)‐shRNA‐CMV‐eGFP‐WPRE vectors into the corpus callosum (Figure S5C). Fourteen days after vector injection, western blotting confirmed that SLC11A1 protein expression was reduced by approximately 50% in the knockdown group (Figure S5A,B). Notably, IF staining for myelin basic protein (MBP) revealed an approximately 50% reduction in the infarct volume in *Slc11a1*‐shRNA–treated mice compared with that in control mice at week 9 post‐stroke (Figure S5D,E). Additionally, *Slc11a1* knockdown increased Caspr expression to levels comparable to those observed in the contralateral hemisphere (Figure S5F). Consistent with these pathological and histological findings, behavioral assessments demonstrated significant functional improvement at week 9 post‐stroke in the *Slc11a1* knockdown group (Figure S5G,H). Moreover, myelin debris was more extensively engulfed and degraded within the lysosomes of activated microglia at weeks 3 and 9 post‐stroke (Figure S5I–L). As expected, *Slc11a1* knockdown effectively reduced the MFI of FerroOrange in lysosomes (Figure S5M,N). In summary, *Slc11a1* knockdown promotes white matter recovery in mice, likely by mitigating iron accumulation within the lysosomes of activated microglia.

### 2.5 Specific Knockdown of *Slc11a1* in Microglia Facilitates White Matter Repair by Inhibiting Microglial Iron Deposition and Increasing Nuclear Transcription Factor EB (TFEB) Expression

**FIGURE 2:**
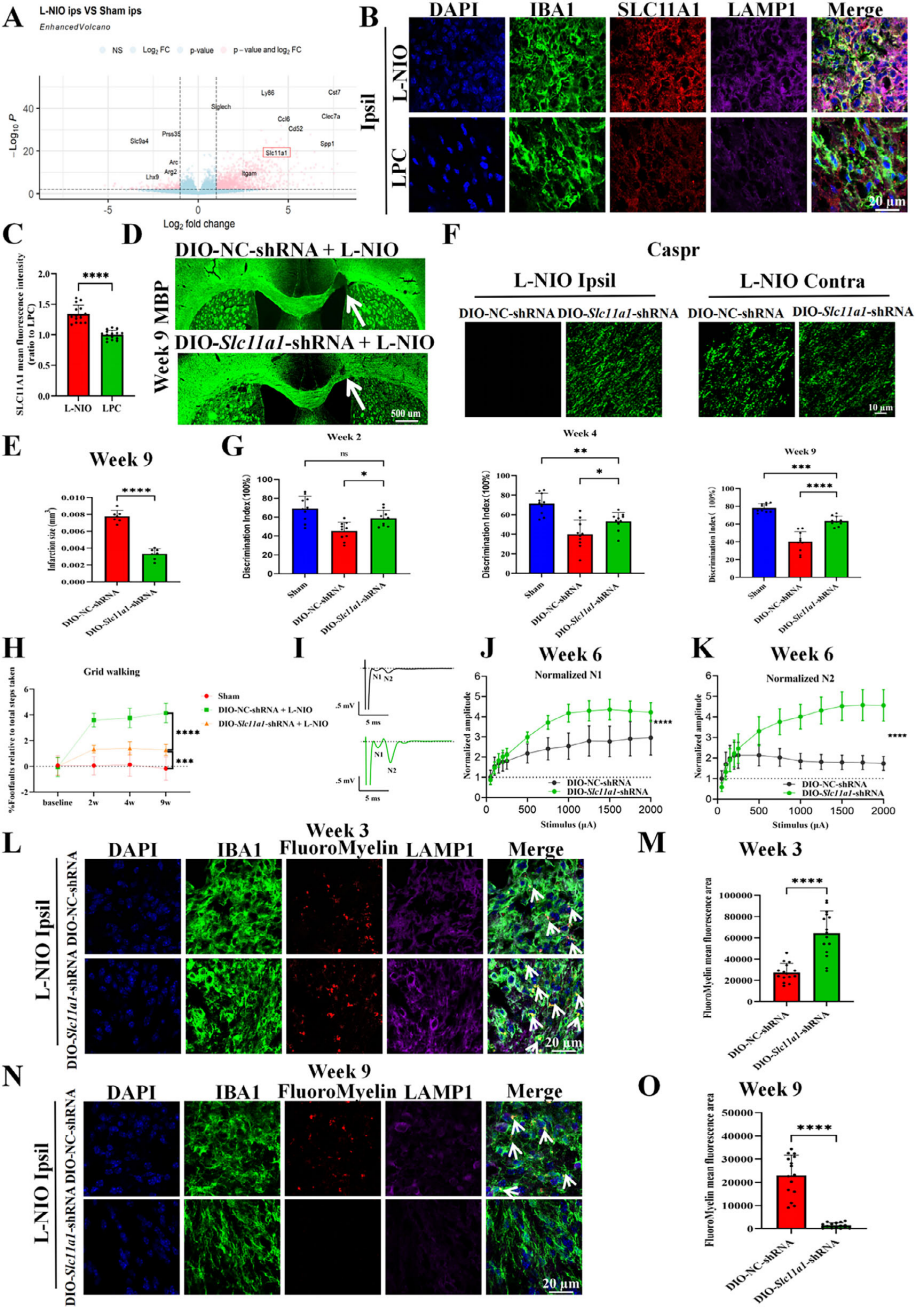
Specific knockdown of *Slc11a1* in the microglia of *Cx3cr1* ^CreERT2^ mice facilitates white matter recovery. (A) Volcano plot presenting significant *Slc11a1* upregulation. (B,C) Representative images presenting SLC11A1 (red), IBA1 (green), and LAMP1 (purple) co‐labeling in each group on day 7 post‐modeling (B; scale bar = 20 µm) and the corresponding quantification (C; each dot represents the average of three fields per brain section, *n* = 3 sections per mouse, N = 5 mice per group; two‐tailed unpaired Student's *t*‐test with Welch's correction; 95% *CI*[‐0.4280, ‐0.2567]). (D,E) MBP IF analysis presenting infarct lesions (D; scale bar = 500 µm) and the corresponding quantification (E; N = 7 per group; two‐tailed unpaired Student's *t*‐test; 95% *CI*[‐0.005227, ‐0.003661]). White arrows indicate lesions. (F) Representative images of Caspr expression in various groups (three fields per brain section, n = 3 brain sections per mouse, N = 5 mice per group; scale bar = 10 µm). (G,H) Novel object recognition (G) and grid walking tests (H) at weeks 2, 4, and 9 post‐stroke in sham, DIO‐*Slc11a1*‐shRNA–treated, and control groups (N = 10 per group; one‐way ANOVA followed by Tukey's post hoc test; G: DIO‐*Slc11a1*‐shRNA vs DIO‐NC‐shRNA: Week 2 95% *CI*[‐25.18, ‐1.809], Week 4 95% *CI*[‐26.23, ‐0.2408], Week 9 95% *CI*[‐31.93, ‐14.62]; H: DIO‐*Slc11a1*‐shRNA vs DIO‐NC‐shRNA:Week 9 95% *CI*[2.058, 3.663]). (I) Sample traces of CAPs under 500‐µA stimulation. J, K) N1 and N2 amplitudes evoked by stimulation at 50, 100, 150, 200, 250, 500, 750, 1000, 1250, 1500, 1750, and 2000 µA; the presenting values were normalized to the means obtained with 50‐µA stimulation. DIO‐NC‐shRNA, n = 10/3 (10 slices from 3 animals); DIO‐*Slc11a1*‐shRNA, *n* = 10/3; two‐way ANOVA; N1:95% *CI*[‐1.243, ‐0.4194]), N2:95% *CI*[‐1.741, ‐0.8369]). L–O) Representative images of DAPI (blue), IBA1 (green), FluoroMyelin (red), and LAMP1 staining (purple) at weeks 3 (L; scale bar = 20 µm) and 9 (N; scale bar = 20 µm) post‐stroke (L, N; white arrows indicate colocalization of FluoroMyelin with LAMP1 and IBA1) and the corresponding quantification (M, O; each dot represents the average of three fields per brain section, *n* = 3 brain sections per mouse, N = 5 mice per group; two‐tailed unpaired Student's *t*‐test with Welch's correction; Week 3 95% *CI*[24505, 49182], Week 9 95% *CI*[‐26401, ‐16671]). Data are presented as the mean ± SEM. *p < 0.05; ***p < 0.001; ****p < 0.0001; ns, not significant.

As SLC11A1 is mainly expressed in microglia within the CNS and its knockdown promotes white matter recovery in C57BL/6 mice through a mechanism involving iron transport, we sought to specifically reduce *Slc11a1* expression in the microglia of *Cx3cr1* ^CreERT2^ mice to determine whether microglia‐specific knockdown promotes white matter recovery. Based on prior research [29], a lentiviral vector was designed to co‐express *Slc11a1*‐shRNA and EGFP in a Cre recombinase‐dependent manner (Figure S6A). We then stereotactically injected either the lentiviral vectors or the control vectors into the corpus callosum of the *Cx3cr1* ^CreERT2^ mice. Following the experimental timeline, tamoxifen was administered for 5 consecutive days beginning on day 4 after lentiviral vector injection (Figure S6B). To assess the targeting efficiency and specificity of the vector in microglia within the CNS, the lentivirus (LV) was injected into the corpus callosum of *Cx3cr1* ^CreERT2^: *Rosa* ^tdT/+^ microglial lineage‐tracing mice. EGFP was predominantly expressed in microglia (tdT‐positive cells), implicating the specific expression of *Slc11a1* in microglia (Figure S6C). Additionally, western blotting confirmed that SLC11A1 protein expression was reduced by approximately 50% in *Cx3cr1* ^CreERT2^ mice (Figure S6D,E). We next investigated the effect of microglia‐specific *Slc11a1* knockdown on WMS recovery. Mice in the *Slc11a1* knockdown group exhibited decreased infarct volumes (Figure 2D,E) and increased Caspr expression (Figure 2F) at week 9 post‐stroke relative to the findings in control mice. Functionally, the mice with microglial specific knockdown of *Slc11a1* exhibited substantially improved neurological deficits (Figure 2G,H). Consistent with the behavioral performance, specific knockdown of *Slc11a1* significantly enhanced both the first and second negative peaks (N1 and N2) of compound action potentials (CAPs) evoked by step‐up stimulus (Figure 2I–K), indicating that *Slc11a1* ablation is beneficial for the repair of heavily myelinated, thinly myelinated, and unmyelinated axons in L‐NIO models. Confocal microscopy revealed that compared to the microglia of control mice, the microglia of the mice with microglial specific knockdown of *Slc11a1* engulfed more myelin debris at 3 weeks post‐WMS and contained less engulfed myelin debris at 9 weeks post‐WMS, suggesting that *Slc11a1* knockdown promoted microglial uptake and degradation of myelin debris following WMS (Figure 2L–O).

**FIGURE 3:**
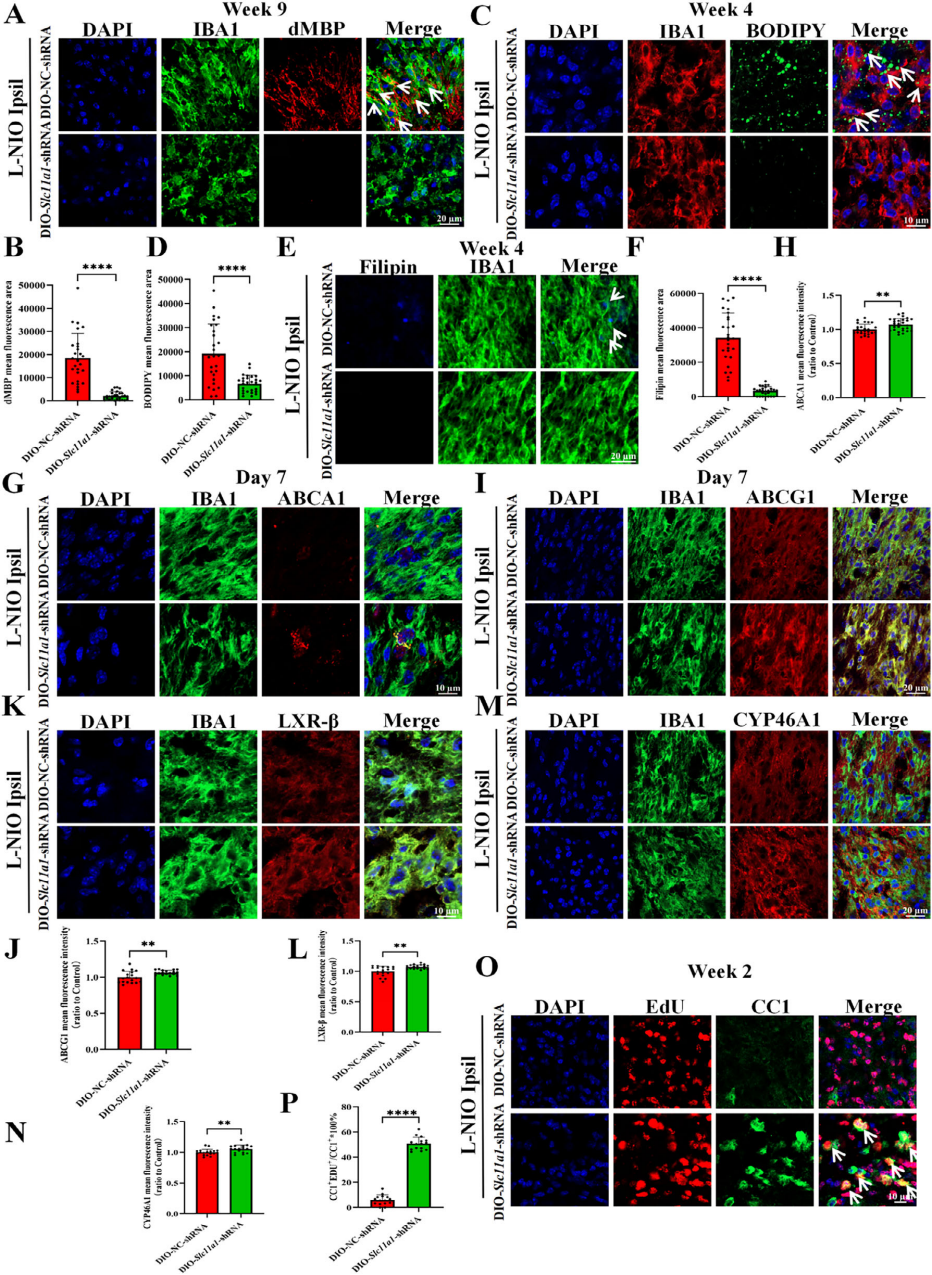
Specific *Slc11a1* knockdown in the microglia of *Cx3cr1* ^CreERT2^ mice facilitates myelin debris clearance. (A,B) Representative images of DAPI (blue), IBA1 (green), and dMBP (red) staining at week 9 post‐stroke (A; scale bar = 20 µm; white arrows indicate colocalization of IBA1 with dMBP) and the corresponding quantification (B; each dot represents a field, three fields per brain section, *n* = 3 brain sections per mouse, N = 3 mice per group; two‐tailed unpaired Student's *t*‐test with Welch's correction; 95% *CI*[‐20648, ‐12031]). (C,D) Representative images of IBA1 (red) and BODIPY (green) co‐labeling in each group at week 4 post‐modeling (C; scale bar = 10 µm; white arrows indicate colocalization of IBA1 with BODIPY) and the corresponding quantification (D; each dot represents a field, three fields per brain section, *n* = 3 brain sections per mouse, N = 3 mice per group; two‐tailed unpaired Student's *t*‐test with Welch's correction; 95% *CI*[‐17525, ‐7447]). (E,F) Representative images of filipin (blue) and IBA1 (green) co‐labeling in each group at week 4 post‐modeling (E; scale bar = 20 µm; white arrows indicate colocalization of IBA1 and filipin) and the corresponding quantification (F; each dot represents a field, three fields per brain section, n = 3 brain sections per mouse, N = 3 mice per group; two‐tailed unpaired Student's *t*‐test with Welch's correction; 95% *CI*[‐36598, ‐25069]). (G,H) Representative images of IBA1 (green) and ABCA1 (red) co‐labeling in each group on day 7 post‐modeling (G; scale bar = 10 µm) and the corresponding quantification (H; each dot represents a field, three fields per brain section, *n* = 3 brain sections per mouse, N = 3 mice per group; two‐tailed unpaired Student's *t*‐test; 95% *CI*[‐0.02628, 0.1172])). (I–N) Representative images of DAPI (blue), ABCG1 (red), LXR‐β (red), CYP46A1 (red), and IBA1 (green) staining on day 7 after L‐NIO modeling (K, scale bar = 10 µm; I, M; scale bar = 20 µm) and the corresponding quantification (J, L, N; each dot represents a field, two fields per brain section, *n* = 3 brain sections per mouse, N = 3 mice per group; j, l, two‐tailed unpaired Student's *t*‐test with Welch's correction; N, two‐tailed unpaired Student's *t*‐test; ABCG1 95% *CI*[0.02124 to 0.1070], LXRβ 95% *CI*[0.03047, 0.1157], CYP46A1 95% *CI*[0.01543, 0.09218]). O, P) Representative images of OPC differentiation into mature OLs on day 14 post‐stroke (O; scale bar = 10 µm) and the corresponding quantification (P; each dot represents the average of three fields per brain section, *n* = 3 brain sections per mouse, N = 5 mice per group; two‐tailed unpaired Student's *t*‐test; 95% *CI*[41.45, 48.37]). White arrows indicate colocalization of EdU and CC1. Data are presented as the mean ± SEM. **p < 0.01; ****p < 0.0001; ns, not significant.

To further verify that microglial knockdown of *Slc11a1* enhanced myelin debris clearance, we used degraded myelin basic protein (dMBP) as another marker of myelin debris. In line with the aforementioned results, *Slc11a1* knockdown in microglia enhanced their ability to clear myelin debris at week 9 post‐stroke (Figure 3A,B). Because myelin fragments are rich in lipids and cholesterol, we subsequently examined deposition of cholesterol and lipid droplets in each group. BODIPY (lipid droplet marker) and filipin (cholesterol marker) staining [30] revealed substantial lipid droplet and cholesterol deposition in the control group at week 4 after L‐NIO modeling, whereas no obvious deposition was observed in the in the mice with microglial specific knockdown of *Slc11a1* (Figure 3C–F). Based on the filipin staining results, bioinformatics analysis (Figure S1B; upregulation of cholesterol metabolism pathway), and previous research [17], cholesterol metabolism is a crucial step in myelin debris clearance and myelin repair. Therefore, we further explored the expression profiles of several key mediators involved in cholesterol degradation or efflux, such as ATP‐binding cassette transporter A1 (ABCA1), ATP‐binding cassette subfamily G member 1 (ABCG1), cytochrome P450, family 46, subfamily A, polypeptide 1 (CYP46A1), and liver X receptor β (LXR‐β) [17, 31, 32]. IF staining and qPCR demonstrated the specific *Slc11a1* knockdown in microglia increased the expression of ABCA1, ABCG1, CYP46A1, and LXR‐β on day 7 post‐modeling (Figure 3G–N; Figure S6F). Collectively, *Slc11a1* knockdown promotes the degradation and clearance of myelin debris, thereby facilitating myelin repair.

As the differentiation of OPCs into mature OLs represents a marker of white matter recovery, we examined the involvement of SLC11A1 in oligodendrogenesis by evaluating the colocalization of 5‐ethynyl‐2′‐deoxyuridine (EdU), a marker of proliferating cells, and CC1, a marker of mature OLs. The results indicated that EdU(+) cells constituted approximately 50% of CC1(+) cells in the mice with microglial specific knockdown of *Slc11a1*, compared with 5.94% in the control group (Figure 3O,P).

**FIGURE 4:**
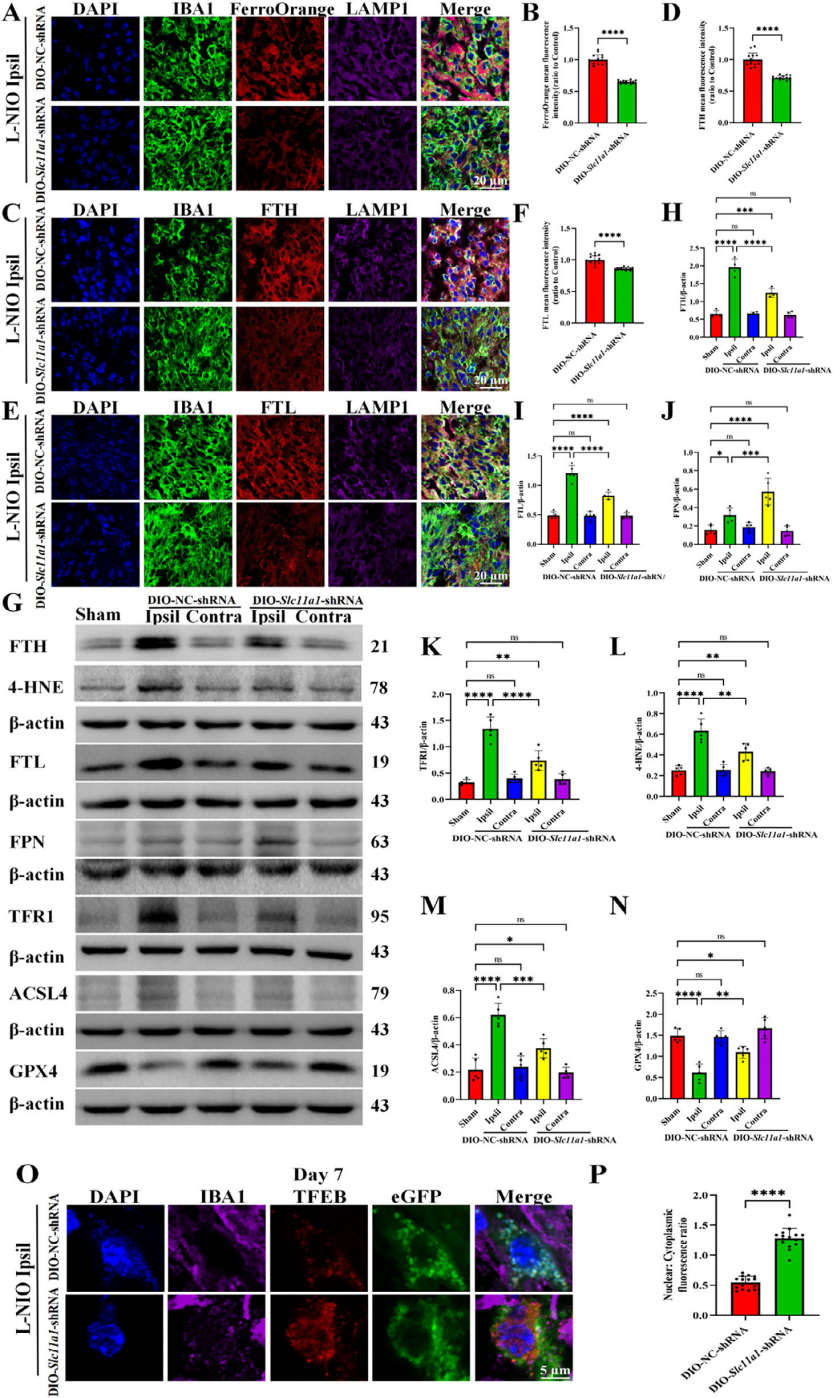
Microglia‐specific *Slc11a1* knockdown in *Cx3cr1* ^CreERT2^ mice mitigates iron accumulation within lysosomes and increases nuclear TFEB expression. (A–F) Representative images of DAPI (blue), IBA1 (green), FerroOrange (red, Fe^2+^), FTH (red, Fe^3+^), FTL (red, Fe^3+^), and LAMP1 (purple) staining on day 7 post‐stroke (A, C, E; scale bar = 20 µm) in the DIO‐*Slc11a1*‐shRNA–treated and control groups and the corresponding quantification (B, D, F; each dot represents the average of three fields per brain section, *n* = 3 brain sections per mouse, N = 5 mice per group; two‐tailed unpaired Student's *t*‐test with Welch's correction; FerroOrange 95% *CI*[‐0.3961, ‐0.3072], FTH 95% *CI*[‐0.3483, ‐0.2313], FTL 95% *CI*[‐0.1797, ‐0.1033]). (G–N) Representative immunoblot bands illustrating the levels of FTH, FTL, FPN, TFR1, 4‐HNE, ACSL4, and GPX4 in the DIO‐*Slc11a1*‐shRNA–treated and control groups (G; FTH, FTL, and 4‐HNE assessed on day 7 post‐stroke; ACSL4, TFR1, and GPX4 assessed on day 4; FPN assessed on day 2) and the corresponding quantification (H–N; FTH, N = 4; others, N = 5; one‐way ANOVA followed by Tukey's post hoc test; DIO‐NC‐shRNA+L‐NIO Ipsil vs DIO‐Slc11a1‐shRNA+L‐NIO Ipsil: FTH 95% *CI*[0.4152, 1.012], FTL 95% *CI*[0.2307, 0.5344], FPN 95% *CI*[‐0.4121, ‐0.09793], TFR1 95% *CI*[0.3275, 0.8777], 4‐HNE 95% *CI*[0.06437, 0.3384], ACSL4 95% *CI*[0.1035, 0.3844], GPX4 95% *CI*[‐0.8389, ‐0.1271]). *β*‐actin served as an internal control. (O) Representative images of TFEB staining on day 7 post‐stroke. DAPI (blue), IBA1 (purple), TFEB (red), and eGFP fluorescence in microglia infected with either DIO‐*Slc11a1* shRNA or DIO‐NC shRNA (green). Scale bar = 5 µm. (P) Quantification of the nuclear‐to‐cytoplasmic fluorescence ratio of TFEB (each dot represents the average of three fields per brain section, *n* = 3 brain sections per mouse, N = 5 mice per group; two‐tailed unpaired Student's *t*‐test; 95% *CI*[0.6279, 0.8381]). Data are presented as the mean ± SEM. *p < 0.05; **p < 0.01; ***p < 0.001; ****p < 0.0001; ns, not significant.

Additionally, IF analysis uncovered a progressive increase in SLC11A1 protein expression in the ipsilateral corpus callosum on day 7 post‐modeling, compared with day 3 in activated microglia (Figure S6G,H). The MFI of FerroOrange, FTH, and FTL in the lysosomes of activated microglia was lower in the mice with microglial specific knockdown of *Slc11a1* than in control mice (Figure 4A‐F). Consistent with these findings, western blotting revealed lower expression of FTH, FTL, TFR1, 4‐HNE, and ACSL4, along with higher expression of FPN and GPX4, in the ipsilateral lesions of the mice with microglial specific knockdown of *Slc11a1* (Figure 4G–N).

According to previous research [33], iron accumulation suppresses the nuclear expression of TFEB, a key regulator of autophagosome–lysosome fusion. Based on this finding, indeed, our results highlighted a higher nuclear‐to‐cytoplasmic fluorescence ratio of TFEB in DIO‐*Slc11a1*‐shRNA–treated mice than in control mice (Figure 4O,P), indicating that *Slc11a1* knockdown promoted TFEB nuclear translocation.

Collectively, these findings suggest that microglia‐specific *Slc11a1* knockdown in *Cx3cr1* ^CreERT2^ mice mitigates lysosomal iron accumulation. This effect enhances the capacity for myelin debris uptake and degradation and promotes TFEB nuclear translocation (activation), which likely mediates the downstream effects of *Slc11a1* knockdown.

### 2.6 *Slc11a1* overexpression Impedes White Matter Recovery in a Repairable LPC‐induced Demyelination Model by Increasing Iron Accumulation

To investigate whether *Slc11a1* overexpression disrupts the reparative process in the LPC model, we constructed pcSLenti‐elongation factor 1‐alpha promoter (EF1)‐eGFP‐CMV‐ *Slc11a1*‐3xFLAG‐WPRE and pcSLenti‐EF1‐eGFP‐CMV‐multiple cloning site (MCS)‐3xFLAG‐WPRE vectors and stereotactically injected them (separately) into the corpus callosum 2 weeks prior to LPC administration. Western blotting confirmed that SLC11A1 protein expression was increased by approximately 50% in the overexpression group (Figure S7A,B). Mice receiving the LV‐*Slc11a1* vector exhibited impaired pathological repair along with significantly larger infarct volumes relative to those in the control group (Figure S7C,D). IF staining also revealed considerably reduced Caspr expression in the ipsilateral lesions of LV‐*Slc11a1–*treated mice (Figure S7E). Consistent with these pathological and histological findings, mice overexpressing *Slc11a1* displayed impaired performance in both the novel object recognition and grid walking tests, indicating persistent neurological deficits and failed white matter recovery (Figure S7F,G). Moreover, abundant myelin debris was detected in lysosomes labeled with LAMP1 in activated microglia in the LV‐*Slc11a1–*treated group at week 3 post‐modeling. Conversely, almost no debris was found in the LV‐NC–treated group (Figure S7H,I), indicating delayed or impeded repair. *Slc11a1* overexpression also led to abundant myelin debris deposited in microglia at 9 weeks after LPC‐induced injury, further suggesting that enhanced SLC11A1 expression inhibits the clearance of myelin debris following WMS (Figure S7J,K). Thus, we concluded that *Slc11a1* overexpression abolished the reparative capacity of demyelinated lesions in the LPC model.

Consistent with our hypothesis and prior findings (Figure 4A–F), LV‐*Slc11a1* administration significantly increased the MFI of FerroOrange, FTH, and FTL, indicating exacerbated iron accumulation in the LV‐*Slc11a1–*treated LPC model (Figures S7L,M and S8A–D). Additionally, FTH, FTL, TFR1, 4‐HNE, and ACSL4 levels were elevated and FPN and GPX4 levels were reduced in the ipsilateral lesions of LV‐*Slc11a1–*treated mice after LPC modeling (Figure S8E–L). These changes collectively led to enhanced iron accumulation.

### 2.7 Single‐Cell RNA Sequencing (scRNA‐seq) Reveals Downstream Molecules Associated with *Slc11a1* Knockdown and Potential Intracellular Signaling Pathways

**FIGURE 5:**
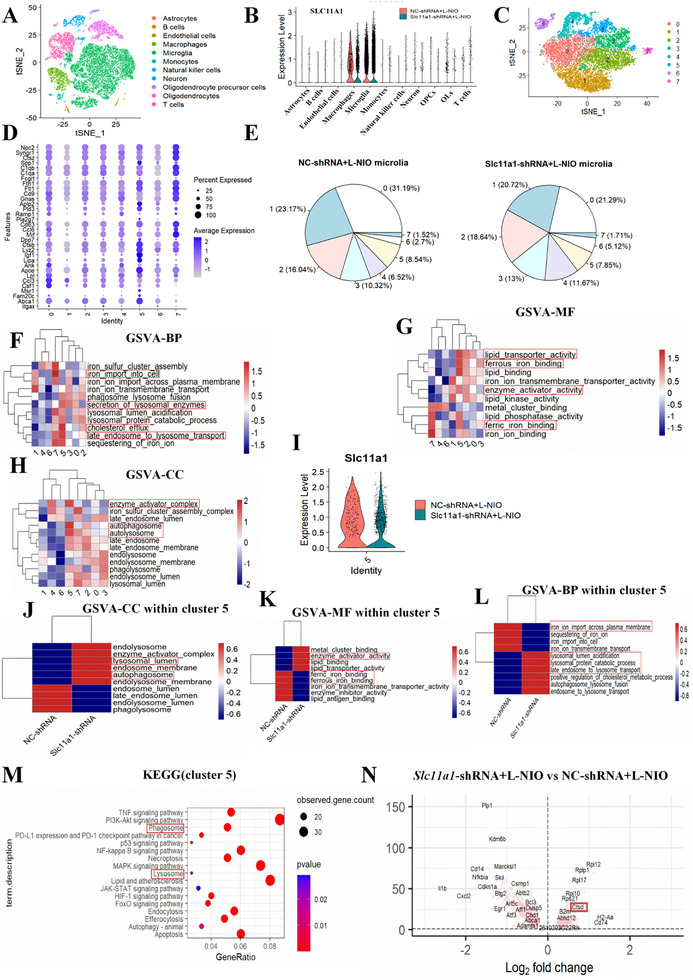
scRNA‐seq reveals downstream molecules of *Slc11a1* and potential intracellular signaling pathways. (A) t‐SNE plot of cells from *Slc11a1*‐shRNA–treated and control mice on day 7 post‐stroke, revealing 11 initial clusters. (B) Violin plot presenting SLC11A1 expression across various cell types. SLC11A1 expression in macrophages showed no difference between the *Slc11a1*‐knockdown group and the control group following WMS (avg_log2FC = −0.135, adjusted *p* = 0.0817), whereas its expression in microglia exhibited a significant difference between the two groups following WMS (avg_log2FC = −0.206, adjusted *p* = 6.05*10^−52^). (C) t‐SNE plot of microglia subdivided into eight clusters. (D) Dot plot displaying selected DEGs for each cluster. (E) Pie chart presenting the proportions of eight microglia subclusters in the *Slc11a1*‐shRNA‐L‐NIO and NC‐shRNA‐L‐NIO groups. (F–H) GSVA based on GO analysis of microglia clusters presenting upregulated and downregulated BP, MF, and CC pathways. (I) Violin plot presenting SLC11A1 expression in the *Slc11a1*‐shRNA–treated and control subgroups within cluster 5 (avg_log2FC = −0.312, adjusted *p* = 0.000426). (J–L) GSVA of cluster 5 highlighting upregulated and downregulated GO pathways in the *Slc11a1*‐shRNA–treated and control subgroups. (M) Bubble plot presenting KEGG pathway enrichment of DEGs between the experimental and control subgroups in cluster 5. (N) Volcano plot illustrating upregulated and downregulated DEGs between the experimental and control subgroups in cluster 5.

Next, we investigated the transcriptional mechanisms by which *Slc11a1* knockdown regulated iron transport and white matter regeneration. Thus, we performed a direct clustering comparison between the *Slc11a1*‐depleted and control groups on day 7 post‐stroke using scRNA‐seq. After filtering and quality control, 23 020 distinct genes were included in the analysis. The data indicated that microglia constituted a large proportion of isolated cells within the ischemic corpus callosum lesion. Based on known cell lineage markers from published literature and the CellMarker database, unsupervised clustering identified 11 initial cell clusters (Figure 5A; Figure S9A,B). Consistent with previous studies [9, 10], SLC11A1 expression was predominantly detected in microglia/macrophages (Figure 5B). However, SLC11A1 expression in macrophages showed no difference between the *Slc11a1*‐knockdown group and the control group following WMS (Figure 5B; macrophages: avg_log2FC = −0.135, adjusted *p* = 0.0817), whereas its expression in microglia exhibited a significant difference between the two groups following WMS (avg_log2FC = −0.206, adjusted *p* = 6.05*10^−52^). Macrophages were further subdivided into five clusters by t‐distributed stochastic neighbor embedding (t‐SNE) and uniform manifold approximation and projection (UMAP) according to the selected differentially expressed genes (DEGs) markers [34] (Figure S9C–E). Notably, SLC11A1 expression across all macrophage subclusters showed no difference between the *Slc11a1*‐knockdown group and the control group (Figure S9F). Collectively, these results suggested that the lentiviral approach used in the study primarily decreased SLC11A1 expression in microglia following WMS.

We next focused on the role of microglia in remyelination; specifically, we functionally subdivided the microglial population into eight distinct clusters (Figure 5C; Figure S9G). Among these, cluster 5 attracted particular interest because of its high expression of genes such as cathepsin B (*Ctsb*), secreted phosphoprotein 1 (*Spp1*), lysosomal acid lipase A (*Lipa*), apolipoprotein E (*Apoe*), and *Abca1* (Figure 5D). These genes are associated with foam‐cell differentiation and lipid storage (*Abca1*), responses to lipoprotein particles (*Apoe*, *Abca1*), lysosomal function (*Lipa*, *Ctsb*), regulation of inflammatory responses (*Apoe*), and potential neuroprotection (*Spp1*) [35, 36]. As these processes contribute to myelin debris uptake and clearance [35], cluster 5 might play an important role in white matter recovery. We then analyzed the proportions of microglia subclusters, with no significant differences noted between the experimental and control groups (Figure 5E). To functionally compare cluster 5 with the other clusters, we performed gene set variation analysis (GSVA) based on Gene Ontology (GO) terms. In the biological process (BP) category, cluster 5 exhibited activation of pathways involved in the secretion of lysosomal enzymes, cholesterol efflux, and late endosome to lysosome transport with enrichment observed in the autolysosome, enzyme activator complex, and autophagosome compartments. The downregulated pathways were involved in iron import into cells (Figure 5F,H). In the molecular function (MF) category, cluster 5 displayed elevated activity of pathways related to lipid transporter activity, ferrous and ferric iron binding, and enzyme activation (Figure 5G).

Given these meaningful findings, further exploration of cluster 5 was essential. Notably, SLC11A1 expression in cluster 5 was substantially lower in *Slc11a1*‐shRNA–treated mice than in controls (Figure 5I; avg_log2FC = −0.312, adjusted *p* = 0.000426). We next conducted a functional comparison within cluster 5 between microglia isolated from *Slc11a1*‐shRNA–treated and control mice. GSVA of the BP category revealed upregulation of pathways related to lysosomal lumen acidification, late endosome to lysosome transport, and lysosomal protein catabolism alongside downregulation of iron ion import across the plasma membrane (Figure 5L). GSVA of the cellular component (CC) category highlighted enrichment in the lysosomal lumen and autophagosome pathways, whereas MF analysis demonstrated upregulation of enzyme activator activity (Figure 5J,K). Strikingly, 1047 DEGs were identified between *Slc11a1*‐shRNA–treated and control lesion samples in cluster 5. Consistent with the GSVA results, Kyoto Encyclopedia of Genes and Genomes (KEGG) pathway enrichment analysis of these DEGs indicated significant enrichment in pathways related to lysosomal function and phagosome (Figure 5M). Additionally, DEGs analysis of microglial cluster 5, comparing the *Slc11a1*‐shRNA–treated groups with control groups, identified *Ctsd* as a potential downstream target of *Slc11a1* knockdown (Figure 5N).

### 2.8 Microglia‐Specific Overexpression of *Ctsd* in *Cx3cr1* ^CreERT2^ Mice Promotes Recovery from Ischemic White Matter Injury

**FIGURE 6:**
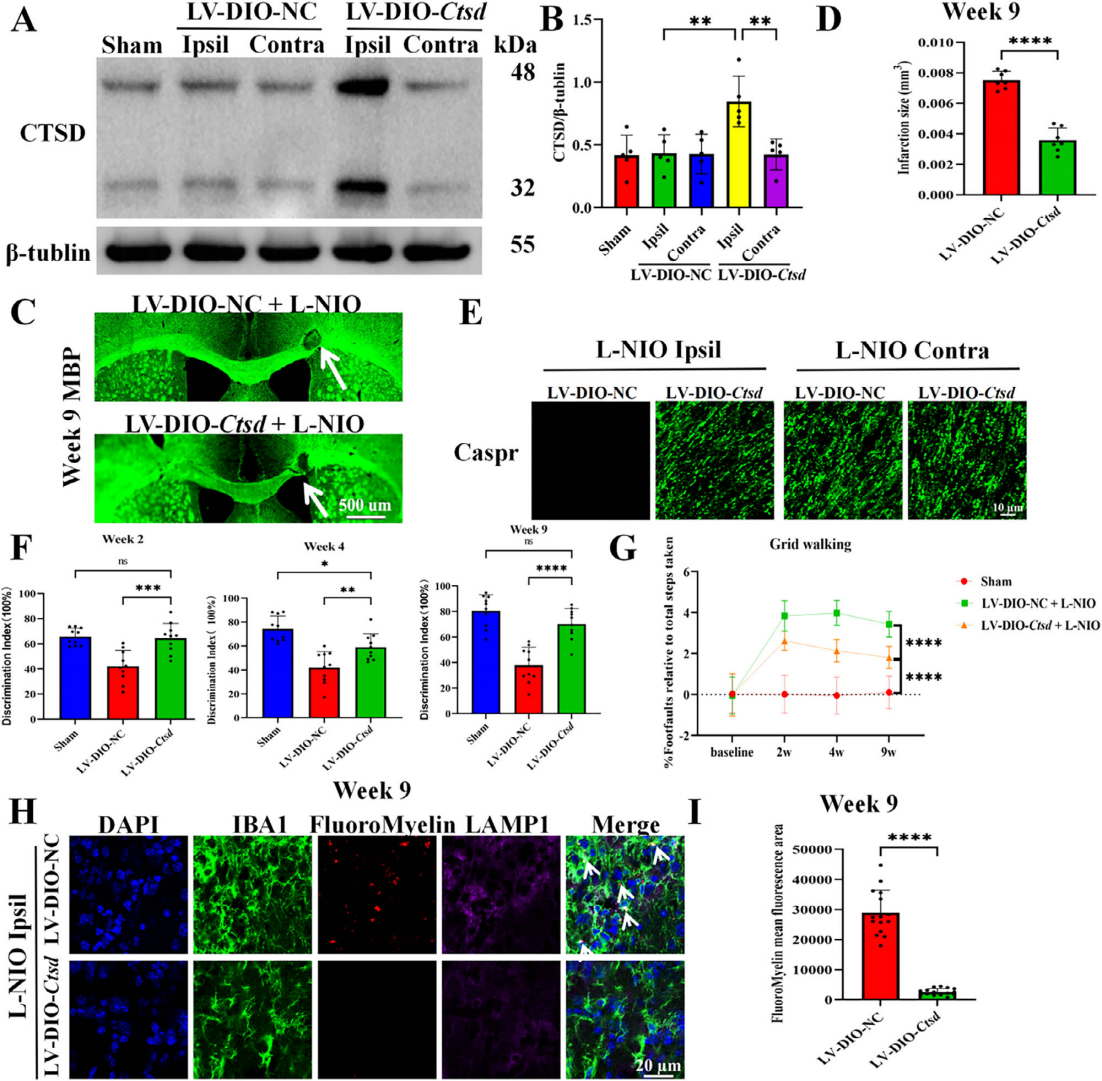
Specific *Ctsd* overexpression in *Cx3cr1* ^CreERT2^ mice promotes ischemic white matter recovery. (A,B) *Ctsd* overexpression efficiency in *Cx3cr1* ^CreERT2^ mice (A) and the corresponding quantification (B; N = 5 per group; one‐way ANOVA followed by Tukey's post hoc test; LV‐DIO‐NC Ipsil vs LV‐DIO‐*Ctsd* Ipsil: 95% *CI*[‐0.7149, ‐0.1106]). *β*‐tubulin served as an internal control. (C,D) Representative images of MBP staining presenting infarct lesions in LV‐DIO‐*Ctsd–*treated and control mice (C; white arrows indicate lesion sites; scale bar = 500 µm) and statistical analysis (D; N = 7 per group; two‐tailed unpaired Student's *t*‐test; 95% *CI*[‐0.004761, ‐0.003128]). (E) IF staining presenting Caspr expression (three fields per brain section, *n* = 3 brain sections per mouse, N = 5 mice per group; scale bar = 10 µm). (F,G) Assessment of neurological deficits by the novel object recognition (F) and grid walking tests (G) at weeks 2, 4, and 9 post‐stroke (N = 10 per group; one‐way ANOVA followed by Tukey's post hoc test; F: LV‐DIO‐*Ctsd*+L‐NIO vs LV‐DIO‐NC+L‐NIO: Week 2 95% *CI*[‐34.32, ‐10.46], Week 4 95% *CI*[‐30.17, ‐3.789], Week 9 95% *CI*[‐46.74, ‐18.05]; G:LV‐DIO‐*Ctsd*+L‐NIO vs LV‐DIO‐NC+L‐NIO:Week 9 95% *CI*[0.8918, 2.359]). (H,I) Representative images of DAPI (blue), IBA1 (green), FluoroMyelin (red), and LAMP1 (purple) staining at week 9 post‐stroke (H; white arrows indicate colocalization of FluoroMyelin with LAMP1 and IBA, scale bar = 20 µm) and the corresponding quantification (I; each dot represents the average of three fields per brain section, *n* = 3 brain sections per mouse, N = 5 mice per group; two‐tailed unpaired Student's *t*‐test with Welch's correction; 95% CI[‐30531, ‐22151]). Data are presented as the mean ± SEM. *p < 0.05; **p < 0.01; ***p < 0.001; ****p < 0.0001; ns, not significant.

As *Ctsd* was identified as a downstream target of *Slc11a1* in microglia by scRNA‐seq, we validated this finding using in vivo experiments. To specifically overexpressed *Ctsd* in the microglia, the lentivirus conditionally overexpressing *Ctsd* in a Cre‐dependent manner (LV‐DIO‐*Ctsd*) or the control lentivirus was injected into the corpus callosum of *Cx3cr1* ^CreERT2^ mice (Figure S6B). Western blotting confirmed that CTSD protein expression was increased by approximately 50% in *Cx3cr1* ^CreERT2^ mice (Figure 6A,B). After LV‐DIO‐*Ctsd* administration, infarct volumes in the corpus callosum were significantly diminished (Figure 6C,D). IF staining revealed that Caspr expression in the ipsilateral lesions of treated mice nearly recovered to the levels in the contralateral hemisphere at week 9 post‐stroke, whereas it remained low in the control group (Figure 6E). Neurological function, as assessed by the novel object recognition and grid walking tests, was substantially improved in *Ctsd*‐overexpressing mice compared with that in control mice (Figure 6F,G). Consistent with these behavioral outcomes, near‐complete clearance of myelin debris was detected in LV‐DIO‐*Ctsd–*treated mice by week 9 post‐stroke (Figure 6H,I).

### 2.9 Microglia‐Specific *Ctsd* Knockdown in *Cx3cr1* ^CreERT2^ Mice Impedes White Matter Recovery in a Repairable LPC‐induced Demyelination Model

As *Ctsd* overexpression promoted WMS recovery, we investigated whether microglia‐specific *Ctsd* knockdown abolished the reparative response in the LPC model. Western blotting confirmed that CTSD protein expression was decreased by approximately 50% in *Cx3cr1* ^CreERT2^ mice receiving lentivirus expressing *Ctsd*‐shRNA in the Cre‐dependent manner (Figure S10A,B). MBP staining revealed minimal pathological recovery of lesions in *Ctsd*‐knockdown mice (Figure S10C,D). IF analysis indicated reduced Caspr expression in the ipsilateral lesions of *Ctsd*‐knockdown mice (Figure S10E). Consistent with the pathological and histological findings, *Ctsd*‐knockdown mice displayed lower scores in the novel object recognition test and a higher number of foot faults in the grid walking test (Figure S10F,G). Additionally, myelin debris remained largely undegraded in the *Ctsd*‐knockdown group, resulting in persistent accumulation within lysosomes (Figure S10H,I). Taken together, these results demonstrate that specific *Ctsd* overexpression in *Cx3cr1* ^CreERT2^ mice promotes WMS recovery, whereas *Ctsd* knockdown impedes recovery in a repairable LPC‐induced demyelination model. These findings further suggest that CTSD functions as a downstream effector of SLC11A1.

### 2.10 *Slc11a1* Knockdown Enhances Lysosomal Myelin Uptake and Degradation by Reducing Iron Accumulation and Promoting Lysosomal Acidification In Vitro

To determine whether *Slc11a1* knockdown exerts similar effects on myelin uptake and clearance in vitro, we cultured primary microglia and infected them with either lentivirus expressing *Slc11a1‐*shRNA or control virus expressing NC‐shRNA for 48 h. Purified myelin labeled with PKH26 (a fluorescent dye) was then added to the cultures, and co‐culture was maintained for either 3.5 or 6 h. IF analysis revealed that the PKH26 mean fluorescence area was significantly larger in *Slc11a1*‐shRNA–treated microglia than in control cells at 3.5 h but significantly lower at 6 h, indicating enhanced uptake followed by accelerated degradation (Figure S11A–F).

**FIGURE 7:**
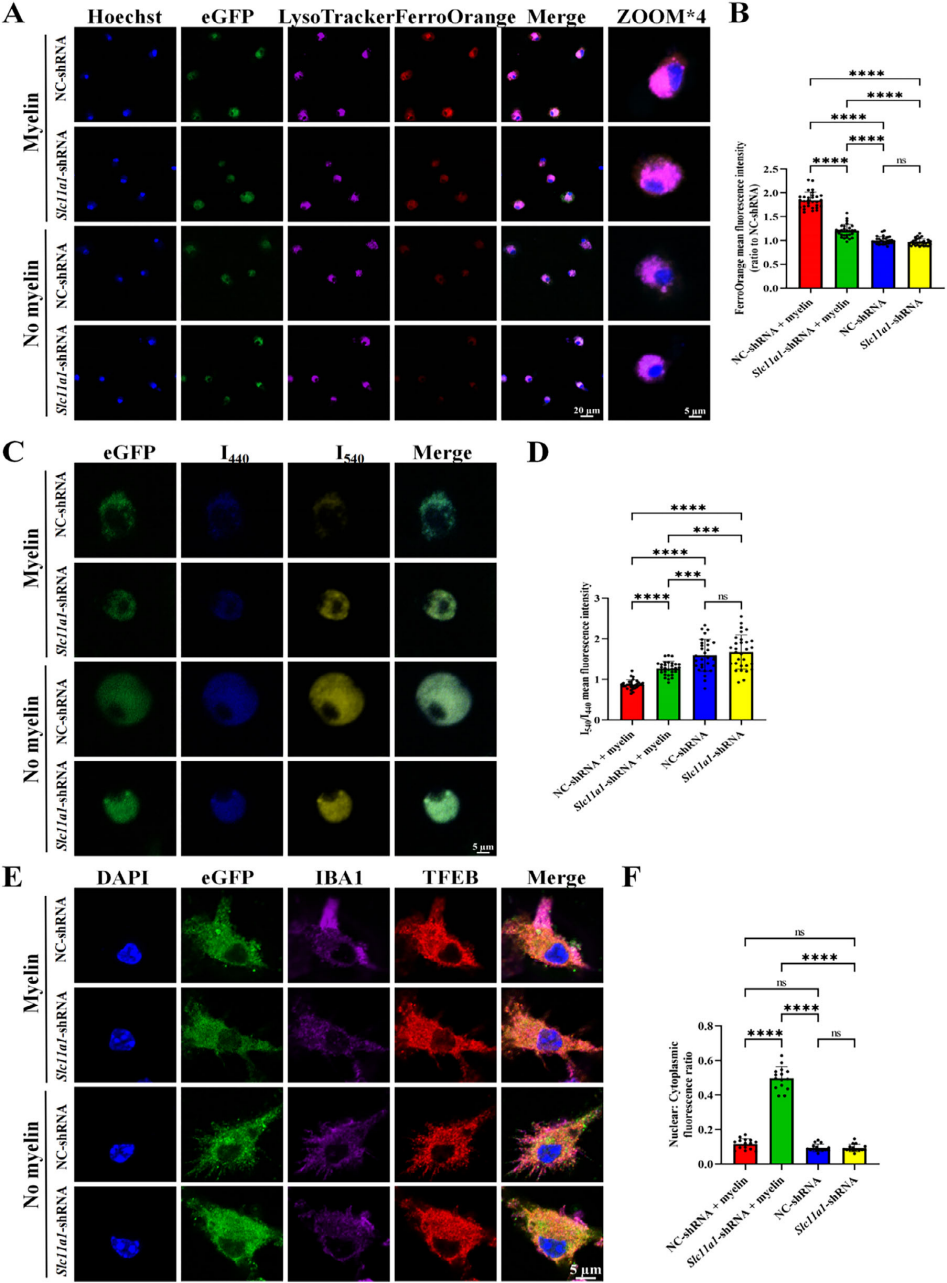
*Slc11a1* knockdown decreases iron accumulation and increases lysosomal lumen acidification in vitro. (A,B) Live‐cell confocal imaging presenting Hoechst (blue), eGFP (green), LysoTracker (purple), and FerroOrange (red, Fe^2+^) staining in *Slc11a1‐*shRNA‐ or NC‐shRNA‐infected primary microglia (A; scale bar = 20 µm, zoom scale bar = 5 µm) and the corresponding quantification (B; three fields per plate, N = 10 plates per group; Welch's ANOVA test followed by Dunnett's T3 post hoc test; NC‐shRNA+myelin vs *Slc11a1*‐shRNA+myelin:95% *CI*[0.5350, 0.7512]). Myelin was added to primary microglia infected with lentivirus for 2 h. (C,D) Representative images of eGFP‐expressing microglia stained with LysoSensor Yellow/Blue probes (C; scale bar = 5 µm) and the corresponding quantification (D; three fields per plate, N = 10 plates per group; Welch's ANOVA test followed by Dunnett's T3 post hoc test; NC‐shRNA+myelin vs *Slc11a1*‐shRNA+myelin:95% *CI*[‐0.4954, ‐0.2923]). Blue fluorescence with an emission peak at 440 nm represents neutral or impaired lysosomes, and yellow fluorescence with an emission peak at 540 nm indicates acidic, functional lysosomes. (E,F) IF staining presenting DAPI (blue), eGFP (green), IBA1 (purple), and TFEB (red) (E; scale bar = 5 µm) and the corresponding quantification of nuclear‐to‐cytoplasmic TFEB fluorescence ratios (F; three fields per plate, N = 5 plates per group; Welch's ANOVA test followed by Dunnett's T3 post hoc test; NC‐shRNA+myelin vs *Slc11a1*‐shRNA+myelin:95% *CI*[‐0.4359, ‐0.3276]). Primary microglia infected with lentivirus were treated with myelin for 3 h before sample collection. Data are presented as the mean ± SEM. ***p < 0.001; ****p < 0.0001; ns, not significant.

We then investigated the effects of *Slc11a1* knockdown on iron and H^+^ transport within primary microglia exposed to myelin. Live‐cell imaging via confocal microscopy revealed that the MFI of FerroOrange in lysosomes, labeled by LysoTracker, was significantly higher in myelin‐treated cells than in untreated cells. Conversely, *Slc11a1* shRNA‐infected primary microglia co‐cultured with myelin displayed significantly lower FerroOrange MFI than myelin‐treated controls (Figure 7A,B). To assess lysosomal acidification, LysoSensor Yellow/Blue probes were used to label lysosomes. Compared with untreated controls, myelin‐treated cells exhibited a significantly lower I_540_/I_440_ ratio, indicating reduced lysosomal acidity. However, in myelin‐treated microglia, infection with *Slc11a1‐*shRNA lentivirus resulted in a substantial increase in I_540_/I_440_, suggesting enhanced lysosomal acidification and a potential reversal of myelin‐induced dysfunction (Figure 7C,D). Given our finding that *Slc11a1* knockdown promotes TFEB nuclear translocation in vivo (Figure 4O,P), we validated this effect in vitro. Primary microglia were infected with either *Slc11a1‐*shRNA or NC‐shRNA lentivirus for 48 h and then exposed to myelin for 3 h. The results demonstrated that *Slc11a1‐*shRNA significantly increased the nuclear‐to‐cytoplasmic fluorescence ratio of TFEB, suggesting enhanced TFEB nuclear translocation (Figure 7E,F).

### 2.11 Identification of a Specific SLC11A1 Antagonist by Molecule Dynamics and Validation In Vitro

**FIGURE 8:**
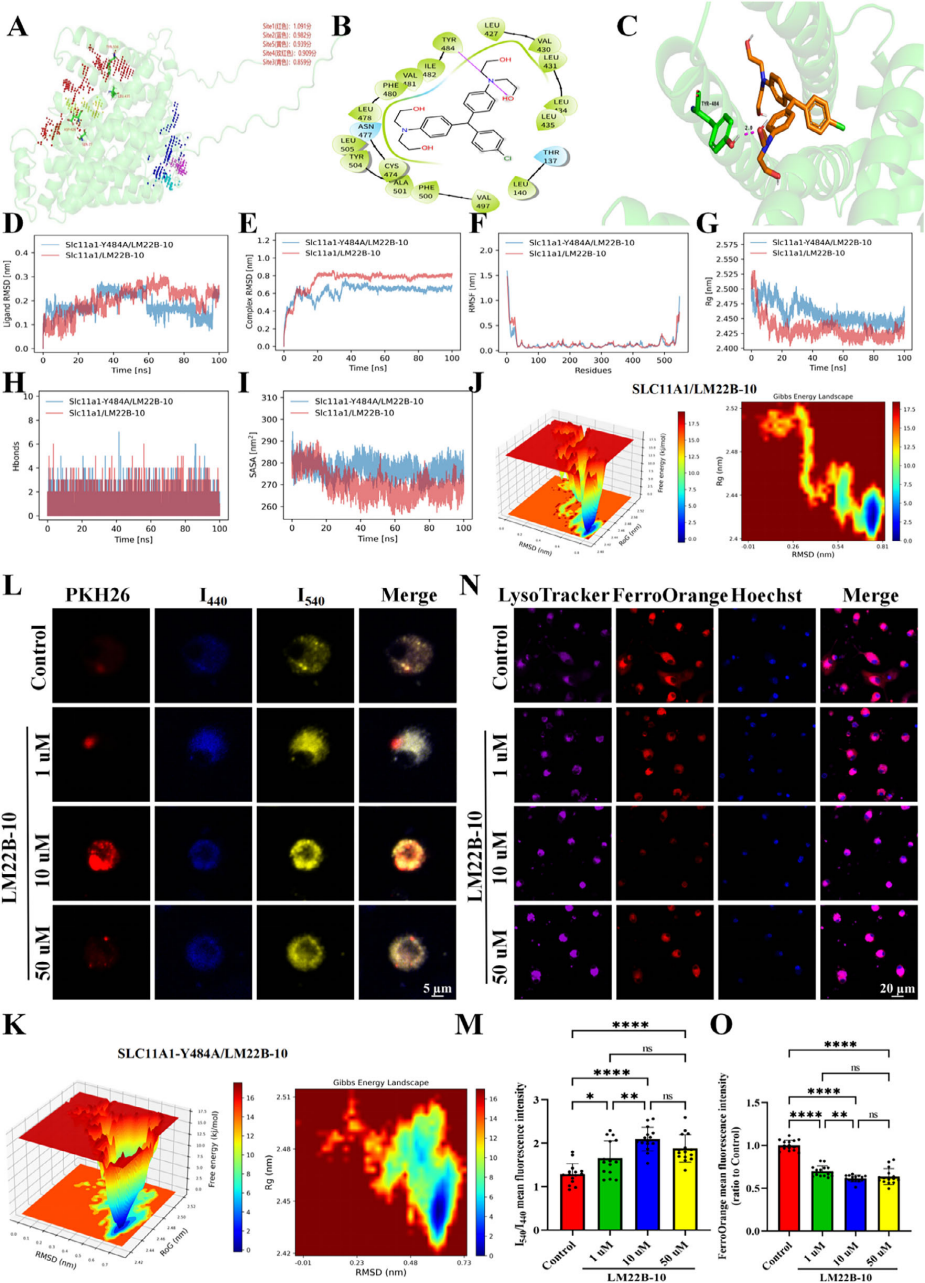
Identification and in vitro validation of a specific SLC11A1 antagonist. (A) 3D structure of SLC11A1 protein and predicted site scores. (B,C) 2D and 3D structural representations of LM22B‐10 and its binding to mouse SLC11A1. (D) Root mean square deviation (RMSD) of LM22B‐10 in the binding pockets of wild‐type and mutant (Y484A) SLC11A1. Larger RMSD values indicate greater fluctuation and lower stability. The RMSD profiles of LM22B‐10 in both SLC11A1 and SLC11A1‐Y484A display similar amplitudes and trends, suggesting no significant difference in ligand mobility between the wild type and the mutant. (E) The RMSD profiles of wild‐type and mutant SLC11A1. Complex RMSD showing the wild‐type complex RMSD stabilizes within the first 20 ns, whereas the mutant (blue) converges around 40 ns and eventually reaches a slightly lower steady value (∼0.7 nm). The wild type (red) shows faster convergence and stable fluctuations, indicating that despite a larger initial deviation from the starting structure, it achieves a more stable bound conformation. (F) Residue flexibility analysis of the root mean square fluctuation (RMSF) distributions for both systems. The main chain regions remain rigid (<0.2 nm), while the N‐ and C‐termini exhibit higher flexibility, consistent with their inherently dynamic nature. (G) Radius of Gyration (Rg) reflecting the overall compactness of the complex. The wild‐type complex (red) rapidly decreases to ∼2.46 nm and remains stable, indicating a more compact structure. The mutant (blue) maintains slightly higher and more variable Rg values, suggesting a looser, less compact architecture. This reduced compactness may weaken ligand binding, aligning with the observed differences in binding free energy. (H) The distribution of hydrogen bonds between the protein and ligand for both the wild‐type and mutant systems, generally fluctuating between 0–6 and most frequently 0–2, indicating no major difference in hydrogen bonding patterns. (I) Solvent accessible surface area (SASA) as a measure of the solvent‐exposed surface area, which correlates with structural compactness. The wild‐type complex shows lower SASA values (∼255–275 nm^2^) during the mid‐to‐late simulation, whereas the mutant exhibits notably higher values (∼270–390 nm^2^). The increased exposure suggests a more relaxed, open conformation in the mutant, further supporting its reduced binding affinity. (J) Free‐energy minimum of the wild‐type and mutant systems, indicating a stable complex conformation and a firmly bound ligand, with the system dominated by a single low‐energy state. (K) The lowest‐energy region is more widely distributed and the energy well is comparatively flat, suggesting increased conformational fluctuations and reduced stability, which may weaken the drug‐binding capacity. (L,M)) Representative images presenting myelin labeled with PKH26 and lysosomes stained with LysoSensor Yellow/Blue probes (L; scale bar = 5 µm) and corresponding quantification (M; three fields per plate, N = 5 plates per group; one‐way ANOVA followed by Tukey's post hoc test; 1 vs 10 µM 95% *CI*[‐0.7430, −0.1359], 10 vs 50 µM 95% *CI*[‐0.09033, 0.5167]). Blue fluorescence (emission at 440 nm) indicates neutral or impaired lysosomes, whereas yellow fluorescence (emission at 540 nm) indicates acidic, functional lysosomes. Primary microglia were co‐treated with various concentrations of LM22B‐10 and PKH26‐labeled myelin for 2 h. The control group consisted of primary microglia treated with PKH26‐labeled myelin alone. (N, O) Representative images of FerroOrange staining after co‐treatment with LM22B‐10 and myelin for 2 h (N; scale bar = 20 µm) and quantification (O; three fields per plate, N = 5 plates per group; Welch's ANOVA test followed by Dunnett's T3 post hoc test; 1 vs 10 µM 95% *CI*[0.02911 to 0.1385], 10 vs 50 µM 95% *CI*[‐0.09748, 0.05425]). Hoechst (blue), FerroOrange (red, Fe^2+^), and LysoTracker (purple) were used to label nuclei, Fe^2+^, and acidic lysosomes, respectively. Data are presented as the mean ± SEM. *p < 0.05; **p < 0.01; ***p < 0.001; ****p < 0.0001; ns, not significant.

Based on the 3D protein structure of SLC11A1, the Sitemap module of Schrödinger software was used to predict potential binding pockets. Five candidate sites, namely sites 1 (1.091), 2 (0.982), 3 (0.939), 4 (0.909), and 5 (0.859), were identified (Figure 8A). Virtual screening was then conducted using the site 1, which had the highest score. Among 200 compounds screened from the MedChemExpress Bioactive Compound Library Plus, LM22B‐10 constituted a promising candidate (Figure 8B,C) because of its known involvement in mitogen‐activated protein kinase and phosphoinositide‐3‐kinase/protein kinase B signaling pathways [37, 38], both of which were enriched in cluster 5 according to KEGG analysis (Figure 5M). Furthermore, LM22B‐10 enhances corneal epithelial healing, increases corneal nerve density, and promotes nerve regeneration in vivo. It also induces neurite outgrowth in vitro [39]. To explore whether LM22B‐10 specifically binds to SLC11A1, we conducted a molecule dynamics experiment. The Y484A mutation in SLC11A1 reduced the conformational stability and free‐energy concentration of the complex, potentially hindering effective binding of LM22B‐10 (Figure 8D–K; Figure S12A,B).

Functionally, considering the role of SLC11A1 in iron and H^+^ transport, we established an in vitro co‐culture model of myelin, LM22B‐10, and primary microglia. LysoSensor Yellow/Blue imaging revealed that I_540_/I_440_ increased as the LM22B‐10 concentration rose to 10 µM. Unexpectedly, this ratio declined at an LB22B‐10 concentration of 50 µM. I_540_/I_440_ peaked in the presence of 10 µM LM22B‐10, significantly differing from that at other concentrations (Figure 8L,M). In parallel, the MFI of FerroOrange was lowest for 10 µM LM22B‐10 (Figure 8N,O). Because LM22B‐10 exerted similar effects on iron and H^+^ transport between lysosomes and the cytoplasm as *Slc11a1* knockdown, we examined whether LM22B‐10 enhanced myelin uptake in BV2 microglia in a concentration‐dependent manner. Quantitative confocal analysis revealed maximal engulfment of PKH26‐labeled myelin in the presence of 10 µM LM22B‐10 (Figure S13A,B). Collectively, these results demonstrated that LM22B‐10 promotes iron export from lysosomes and H^+^ import into lysosomes, thereby enhancing myelin uptake in vitro.

To confirm the role of LM22B‐10 in alleviating iron accumulation within lysosomes in vivo under conditions of brain ischemia, we performed IF staining and quantitative confocal analysis on day 7 post‐stroke. The MFI of FerroOrange, FTH, and FTL was significantly lower in LM22B‐10–treated mice than in control mice (Figure S14A–F). Subsequent western blotting demonstrated that LM22B‐10 administration significantly decreased FTH, FTL, TFR1, 4‐HNE, and ACSL4 levels while increasing FPN and GPX4 levels relative to the control findings (Figure S14G–N). These results demonstrated that LM22B‐10 promotes iron export from lysosomes and attenuates iron accumulation within lysosomes in vivo.

### 2.12 LM22B‐10 Promotes Ischemic White Matter Recovery

Administration of LM22B‐10 significantly diminished the infarct volume, increased Caspr expression, and improved neurological function recovery (Figure S15A–E). Additionally, LM22B‐10 enhanced the lysosomal uptake and degradation of myelin debris (Figure S15F–I). Taken together, LM22B‐10 treatment exhibits comparable effects as *Slc11a1* knockdown in promoting white matter regeneration and represents a potential therapeutic option.

## 3 Discussion

In the present study, we obtained substantial evidence that SLC11A1, localized in microglial lysosomes, mediates iron and H^+^ transport between lysosomes and the cytoplasm both in vitro and in vivo. These findings expand the known functions of SLC11A1 beyond iron and H^+^ metabolism to include its involvement in cellular signaling pathways that regulate WMS recovery. Current evidence indicates that SLC11A1 functions as a bidirectional Fe^2+^ transporter on the membrane of late endosomes and lysosomes, particularly in infectious models [14, 40]. Regarding the potential influence of pH or the cellular redox state, SLC11A1 directionality might differ between infectious and ischemic contexts. In WMS, the directionality of Fe^2+^ transport by SLC11A1 and its role in molecular pathogenesis and repair processes remain unclear. Our investigation indicated that, after WMS, the lysosomal pathway was activated, and the expression of SLC11A1, along with iron metabolism‐ and iron accumulation‐related proteins and compounds (FTH, FTL, TFR1, FPN, ACSL4, and 4‐HNE), continuously increased in the ipsilateral corpus callosum. Their levels gradually declined over time post‐stroke (Figure 2B,C; Figures S1B–I and S2F–M). This time‐dependent expression profile, coupled with the observation that lysosomal iron chelation promotes white matter recovery (Figure 1C–G), supports the conclusion that SLC11A1 and iron accumulation play essential roles in the pathophysiological process of WMS [41]. Excessive iron accumulation occurs after ischemia, and lysosomes act as the primary sites of extracellular iron storage, serving as repositories of labile iron [12]. Ischemia‐induced ROS can compromise lysosomal membrane integrity, leading to Fe^2+^ release and ferroptosis initiation [42]. These findings align with our observations that iron accumulation within microglial lysosomes contributes to the pathogenesis of WMS.

In addition to iron, we observed substantial accumulation of myelin debris in microglial lysosomes as ischemia progressed (Figure S2A). Consistent with prior studies [8, 17, 18], timely and effective clearance of myelin debris in microglia is essential for maintaining a healthy environment that supports myelin repair (Figure S2C–E). However, iron accumulation impairs lysosomal function [43], such as phagocytosis and degradation of myelin debris (Figures S2A–D and S3A–F). Therefore, the reduction of iron accumulation in microglial lysosomes appears to be a critical step for myelin repair.

Although SLC11A1 expression increases under iron‐overloaded conditions in Parkinson's disease models [9], in line with our findings (Figure S1F–I), the mechanism and functional role of SLC11A1 in Fe^2+^ transport remain poorly understood. Mechanistic analyses have indicated that SLC11A1 is either a H^+^/Fe^2+^ cotransporter that depends on the proton gradient [44] or a H^+^/Fe^2+^ antiporter that functions against the proton gradient [45]. Moreover, the directionality of Fe^2+^ transport between lysosomes and the cytoplasm has been a focus of debate. Intriguingly, we found that SLC11A1 transports Fe^2+^ from the cytoplasm into lysosomes, with H^+^ transported in the opposite direction, both in vitro (Figure 7A–D) and in vivo (Figure 4A–F; Figures S7L,M and S8A–D). This finding aligns with prior research [14] and suggests H^+^/Fe^2+^ antiporter activity. Other research indicated that SLC11A1 transports Fe^2+^ from lysosomes to the cytoplasm [46].

Thus far, the role of SLC11A1 in the context of brain ischemia has not been reported. To our knowledge, this is the first study to demonstrate the predominant expression of SLC11A1 in microglial lysosomes and its role as a H^+^/Fe^2+^ antiporter that transports Fe^2+^ from the cytoplasm into lysosomes following WMS both in vivo and in vitro. This novel observation likely reflects the time‐dependent dynamics of SLC11A1 during the molecular pathogenesis of ischemic white matter injury. Functionally, SLC11A1 remains poorly understood. Using the DIO‐Cre approach, we illustrated that genetic *Slc11a1* inhibition reduces iron accumulation in lysosomes (Figure 4A–N), enhances the uptake and clearance of myelin debris (Figure 2L–O and Figure 3A–N; Figure S6F), and promotes OPC differentiation into mature OLs (Figure 4O,P). These effects collectively contribute to white matter repair and neurological functional recovery after stroke (Figure 2D–O and Figure 3A,B). Conversely, *Slc11a1* overexpression increased iron accumulation, impeded myelin debris clearance, and ultimately resulted in failed white matter repair (Figures S7A–K and S7L,M and Figure S8A–L). ScRNA‐seq analysis confirmed our experimental results. GSVA of cluster 5 revealed that *Slc11a1* knockdown facilitated lysosomal lumen acidification and enzyme activator activity, inhibited iron ion import, and reduced iron accumulation (Figure 5J–L). These discoveries agree with previous findings that *Slc11a1* knockdown mitigates liver ischemia–reperfusion injury [13]. Conversely, SLC11A1 reportedly contributes to the degradation of oligomeric α‐synuclein [9]. These contradictory conclusions might have arisen from model‐specific differences in Fe^2+^ transport mechanisms.

Ischemia increases lysosomal pH [47], which impairs lysosomal enzyme activity. Our data suggest that *Slc11a1* knockdown decreases lysosomal pH (Figure 7C,D). Moreover, volcano plot analysis and in vivo experiments identified CTSD as a downstream target of SLC11A1, probably because CTSD is particularly sensitive to lysosomal pH [48, 49]. CTSD is also functionally linked to lysosomal integrity, myelin debris clearance, [11, 48, 50] and cholesterol metabolism [51, 52], all of which contribute to degradation of myelin debris. Notably, we found that *Slc11a1* knockdown alleviated iron accumulation and promoted TFEB nuclear translocation [33], which might mediate CTSD expression both in vitro (Figure 7E,F) and in vivo (Figure 4O,P). On one hand, iron overload induces the production of large amounts of ROS through the Fenton reaction. On the other hand, it compromises lysosomal membrane integrity, resulting in lysosomal alkalization or leakage, which in turn activates mammalian target of rapamycin complex 1 (mTORC1). mTORC1 phosphorylates TFEB, causing it to be retained in the cytoplasm and preventing its nuclear translocation. This in turn leads to the downregulation of lysosomal enzymes such as CTSD and CTSB and thereby impairs lysosomal degradation function [53, 54, 55]. Hence, we hypothesized that *Slc11a1* depletion increases the acidity within microglial lysosomes, reduces iron accumulation, promotes TFEB nuclear translocation, and consequently enhances lysosomal biogenesis and CTSD expression. Although our result suggests a potential regulatory relationship between TFEB and CTSD in WMS (Figure S16), the exact mechanism (e.g., whether TFEB directly binds to the CLEAR motif in the CTSD promoter) remains to be experimentally validated. Prior studies have established TFEB as a master regulator of lysosomal genes, including CTSD, in other contexts [56]. Future work involving TFEB‐specific perturbations (e.g., siRNA or CRISPR) in our model would be necessary to confirm this interaction. Although CTSD activation is likely a principal downstream mechanism after genetic *Slc11a1* inhibition, we cannot exclude the possibility that other intracellular signaling pathways also contribute to white matter recovery.

Our findings have important translational implications, specifically introducing a novel molecular target and therapeutic concept for preventing and treating WMS. In this study, we identified LM22B‐10 as a potential SLC11A1 antagonist through virtual screening and validated its function in primary microglia and BV2 cells. Specifically, LM22B‐10 enhanced H^+^/Fe^2+^ exchange and improved lysosomal function in a concentration‐dependent manner in vitro. However, its efficacy declined at higher concentrations, likely due to cytotoxic effects that impaired lysosomal function in primary microglia and BV2 cells.

Our molecular dynamics studies demonstrated a strong and favorable binding pose for LM22B‐10 within the putative active site of SLC11A1, characterized by a high predicted binding affinity and key molecular interactions (Figure 8A–K; Figure S12A,B). This provided a strong rational hypothesis for SLC11A1 as the primary target and guided our initial functional experiments.

A key limitation of this study was its inability to directly confirm whether SLC11A1 functions as a H^+^/Fe^2+^ antiporter in vivo because of the technical challenge of measuring lysosomal pH in vivo. Although we addressed this issue through complementary in vitro experiments and GSVA, in vivo validation remains an essential goal of future studies. In addition, a significant limitation of molecular docking is the potential for false positives and the inability to comprehensively assess off‐target effects against the vast proteome. Docking simulations are typically performed against a single, static protein structure, and they cannot account for full protein dynamics, solvation effects, or predict binding to proteins with similar active sites. To directly address this and unequivocally confirm the specificity of LM22B‐10 for SLC11A1, we propose as surface plasmon resonance and CRISPR‐Cas9 knockout experiments.

In conclusion, pharmacological or genetic inhibition of SLC11A1, functioning as a H^+^/Fe^2+^ antiporter on the lysosomal membrane, reduces iron transport from the cytoplasm into lysosomes, attenuates iron accumulation within lysosomes, promotes lysosomal lumen acidification, increases CTSD expression, and enhances lysosomal myelin debris uptake and degradation in microglia. These effects promote white matter recovery and establish SLC11A1 suppression as a promising therapeutic strategy for stroke rehabilitation.

## 4 Experimental Section

### 4.1 Mice

C57BL/6 and *Cx3cr* ^Cre^ERT2 mice (expressing Cre under the control of the *Cx3cr1* promoter in macrophages/microglia) were obtained from Charles River Laboratories (Wilmington, MA, USA) and The Jackson Laboratory (025524, Bar Harbor, ME, USA), respectively. All mice were housed in a specific pathogen‐free‐grade animal facility under temperature‐ and humidity‐controlled conditions with ad libitum access to food and water on a 12‐h/12‐h light/dark cycle. Experimental protocols were approved by the Ethics Committee of Soochow University. Female mice were excluded from the study because of the known neuroprotective effects of ovarian hormones, particularly the role of estrogen as a potent antioxidant and neuroinflammation modulator [57].

### 4.2 In Vivo Lentiviral Injection

To knock down *Slc11a1* or *Ctsd* in C57BL/6 or *Cx3cr1*CreERT2 mice, shRNA target sequences reported in previous studies [50, 58] were used to construct lentiviral vectors. The target sequences were as follows: *Slc11a1*‐shRNA, GCCAACATGTACTTCCTGA; NC‐shRNA: TTCTCCGAACGTGTCACGT, DIO‐*Slc11a1*‐shRNA, GCCAACATGTACTTCCTGA; DIO‐NC‐shRNA: GAAGTCGTGAGAAGTAGAA; and DIO‐*Ctsd*‐shRNA, CCTCTTATCCAGGGTGAGTAT.

For overexpression experiments, mouse *Slc11a1* and *Ctsd* genes were cloned into the following lentiviral vectors: LV*‐Slc11a1*, pcSLenti‐EF1‐EGFP‐CMV‐*Slc11a1*‐3xFLAG‐WPRE; LV‐NC, pcSLenti‐EF1‐EGFP‐CMV‐MCS‐3xFLAG‐WPRE, LV‐DIO*‐Ctsd*: pLenti‐CMV‐DIO‐*Ctsd*‐3xFLAG‐P2A‐EGFP‐WPRE; and LV‐DIO‐NC, pLenti‐CMV‐DIO‐EGFP‐WPRE.

The lentiviruses were designed and constructed by Obio Technology Co. (Shanghai, China). Stereotactic injections were performed as previously described [59]. Briefly, male C57BL/6 mice (6–8 weeks old, 23–25 g) were anesthetized and secured in a stereotaxic frame. After midline scalp incision, the skull surface was exposed, and a 1.0‐mm‐diameter burr hole was drilled into the left hemisphere at the designated injection site. A 10.0‐µL microsyringe was inserted using the following coordinates relative to bregma: anterior/posterior, +0.8 mm; medial/lateral, −1.2 mm; and dorsal/ventral, −1.9 mm. Lentiviruses (2.0 µL; *Slc11a1*‐shRNA, NC‐shRNA, DIO‐*Ctsd*‐shRNA, DIO‐*Slc11a1*‐shRNA, DIO‐NC‐shRNA, LV*‐Slc11a1*, LV‐NC, LV‐DIO*‐Ctsd*, or LV‐DIO‐NC) or saline (2.0 µL) was injected into wild‐type or Cre‐dependent mice at a rate of 0.1 µL min^−1^. After injection, the microsyringe was kept in place for 5 min to prevent backflow. All mice underwent baseline grid walking tests after lentiviral or saline microinjection. Mice without detectable deficits were included in subsequent experiments.

### 4.3 Surgical Procedures

Two weeks after lentiviral or saline injection, corpus callosum lesions were induced as previously described [2, 60]. After mice had been successfully anesthetized, WMS lesions were induced via stereotaxic injections of the endothelial nitric oxide synthase inhibitor L‐NIO (400600‐20MG, Sigma–Aldrich, St. Louis, MO, USA) into the lentivirus injection site. L‐NIO was diluted in saline to a final concentration of 27 mg mL^−1^, and 0.5 µL were injected into the corpus callosum at a rate of 0.07 µL min^−1^. Repairable demyelinating lesions were induced via stereotactic injections of LPC (L4129‐25MG, Sigma–Aldrich) diluted in saline to a final concentration of 5 mg mL^−1^. Then, 2.0 µL of LPC were injected into the corpus callosum at a rate of 0.1 µL min^−1^. Sham lesions were induced by injecting an equivalent volume of saline. After each injection, the glass microsyringe was kept in place for 5 min to prevent backflow.

### 4.4 Drug Administration

DFO (HY‐B0988, MedChemExpress, Monmouth Junction, NJ, USA) was dissolved in saline. Immediately after L‐NIO injection, DFO (or an equivalent volume of normal saline) was administered intraperitoneally at 150 mg kg^−1^ day^−1^ for 14 consecutive days as previously described [22, 61]. For in vivo experiments, LM22B‐10 (HY‐104047, MedChemExpress) was first dissolved in PEG300 (HY‐Y0873, MedChemExpress) and then diluted with saline at a 1:1 ratio. Immediately after L‐NIO injection, LM22B‐10 was administered intraperitoneally at 50 mg kg^−1^ day^−1^ for 1 month as previously described [62]. For in vitro experiments, 1 mg of LM22B‐10 was dissolved in 2.0618 mL of dimethyl sulfoxide to obtain a 1‐mM stock solution, which was then diluted in culture medium at ratios of 1:1000, 1:100, and 1:20 to achieve final concentrations of 1, 10, and 50 µM, respectively. Male *Cx3cr*CreERT2 and *Cx3cr1*CreERT2:*Rosa*tdT/+ mice received intraperitoneal tamoxifen (T137974‐5G, Aladdin, Riverside, CA, USA) at 100 mg kg^−1^ day^−1^ for 5 consecutive days beginning 3 days after lentiviral injection. Tamoxifen was dissolved in corn oil (C805618‐500ML, Macklin Biochemical, Shanghai, China) at a concentration of 10 mg mL^−1^ and protected from light during preparation and administration. Control mice received an equivalent volume of corn oil.

### 4.5 Novel Object Recognition Test

The novel object recognition test was performed as previously reported [63, 64] with modifications to assess short‐term memory at weeks 2, 4, and 9 post‐modeling. The test comprised three stages: habituation, object familiarization, and the novel object recognition testing phase. To prevent odor interference and information transfer between groups, mice were housed in separate cages according to their experimental group throughout all stages. During the first stage (habituation), mice were individually placed in a clean, white, open‐field cage (50 × 50 × 50 cm^3^) for 10 min. After each session, the cage and environment were disinfected using 30% ethanol solution. After 3 consecutive days of habituation, the second and third stages were performed on the fourth day. During the second stage (object familiarization), each mouse was placed in the cage and allowed to explore two identical objects for 5 min. After completing this stage, each mouse was labeled in order of testing. One hour later, the third stage (novel object recognition test) was performed. In this phase, one of the original objects was replaced with a novel object placed at the same location as during the familiarization stage, and the other familiar object remained in its original position. Mice were again allowed to explore the objects for 5 min. The novel object recognition test was recorded by video and analyzed using ANY‐maze software (ANY‐maze, Wood Dale, IL, USA). The apparatus, including the cage, objects, and surrounding environment, was disinfected with 30% ethanol between trials to eliminate odors. Cognitive function was quantified using the recognition index (RI), calculated as follows: RI = (time exploring the novel object/time exploring both objects) × 100%.

### 4.6 Grid Walking Test

The grid walking test was used to assess motor coordination and balance at baseline and at weeks 2, 4, and 9 post‐modeling and performed with modifications based on previously described protocols [65, 66]. Briefly, each mouse was placed individually on an elevated wire grid (approximately 2 m high) featuring 1.5 × 1.5 cm^2^ square openings and allowed to walk freely for 5 min per session over 3 consecutive days to minimize anxiety and permit acclimation to the apparatus. Formal testing was performed on day 4. Each mouse walked on the grid for 5 min, during which the total number of steps (including both correct and faulty steps) and foot faults for each limb were recorded. Foot fault asymmetry was calculated using the following formula: [(foot faults of right limb − foot faults of left limb)/total number of steps] × 100%.

### 4.7 CAP Recording

Mice were deeply anesthetized using isoflurane and subjected to cardiac perfusion with ice‐cold oxygen‐saturated (95% O_2_ and 5% CO_2_) cutting solution (2.5 mM KCl, 1.25 mM NaH_2_PO_4_, 25 mM NaHCO_3_, 0.5 mM CaCl_3_, 7 mM MgSO_4_, 210 mM sucrose, 10 mM d‐glucose, and 1.3 mM Na‐ascorbate; all from Sigma–Aldrich). The brain was then removed and sectioned into 400‐µm coronal sections using a vibratome (VT‐1000S, Leica Biosystems, Nussloch, Germany) while submersed in ice‐cold oxygen‐saturated cutting solution. Slices were incubated for 20 min at 34°C, followed by incubation for 1 h at room temperature in artificial cerebrospinal fluid (ACSF, 119 mM NaCl, 2.5 mM KCl, 2.5 mM CaCl_2_ 1.3 mM, MgSO_4_, 26.2 mM NaHCO_3_, 1 mM NaH_2_PO_4_, and 11 mM d‐glucose; continuously bubbled with 95% O_2_/5% CO_2_). Slices containing the hippocampus were used for electrophysiological recordings of CAP. A concentric bipolar stimulation electrode (CBBRC75, FHC, Bowdoin, ME, USA) and a recording electrode (filled with ACSF, 1–2 MΩ resistance) were placed 2 mm apart on the corpus callosum of the same hemibrain. For CAP recording, the stimulating and recording electrodes were placed in the hemisphere of the stroke core, with a distance of 200–300 µm between them. Field potentials recorded via the recording electrode were routed through a Multiclamp 700B amplifier and a Digidata 1550 data acquisition system (both from Molecular Devices, San Jose, CA, USA), filtered at 2 kHz, and digitized at 50 kHz.

### 4.8 Western Blotting

Mice were sacrificed at designated time points (12 h, 24 h, 2 days, 4 days, 7 days, and 14 days post‐modeling and 14 days post‐lentiviral injection). Brains were quickly removed, and lesions in the corpus callosum were dissected on ice using sterile blades. Primary microglia or brain tissue samples were homogenized in RIPA lysis buffer (WB3100, NCM Biotech, Newport, RI, USA) supplemented with protease (FD1001, Fudebio‐tech, Hangzhou, China) and phosphatase (FD1002, Fudebio‐tech) inhibitor cocktails for 2 h on ice. Samples were centrifuged at 13 200 rpm for 30 min at 4°C. The resulting supernatants were collected for immediate use or stored at −20°C for subsequent analysis. Protein concentrations were measured using a bicinchoninic acid protein assay kit (P0012, Beyotime, Haimen, China). Equal amounts of protein from each sample were mixed with 5× loading buffer and heated at 99°C for 8 min. Proteins were separated by electrophoresis on 10% sodium dodecyl sulfate‐polyacrylamide gel electrophoresis gels and transferred to polyvinylidene difluoride membranes (IPVH00010, MilliporeSigma, Burlington, MA, USA). Membranes were blocked with 10% non‐fat milk diluted in 1× Tris‐buffered saline plus Tween (TBST) for 1 h at room temperature (20°C). Next, membranes were washed three times with 1× TBST for 5 min each at room temperature. Membranes were then incubated overnight at 4°C with the following primary antibodies: anti‐TFR1 (13‐6800, 1:3000, Thermo Fisher Scientific, Waltham, MA, USA), anti‐4‐HNE (MA5‐27570, 1:1000, Thermo Fisher Scientific), anti‐FTL (ab109373, 1:5000, Abcam, Cambridge, UK), anti‐FTH (ab183781, 1:5000, Abcam), anti‐FACL4 (ab155282, 1:5000, Abcam), anti‐GPX4 (ET1706‐45, 1:10,000, HUABio, Woburn, MA, USA), anti‐SLC40A1 (ER1916‐80, 1:1000, HUABio), anti‐cathepsin D (ab75852, 1:5000, Abcam), anti‐SLC11A1/NRAMP1 (ANT‐201, 1:1000, Alomone, Jerusalem, Israel), anti‐β‐actin (FD0060, 1:25,000, Fudebio‐tech), and anti‐β‐tubulin (FD0064, 1:25,000, Fudebio‐tech). Membranes were subsequently washed three times with 1× TBST for 5 min each prior to incubation with horseradish peroxidase (HRP)‐conjugated secondary antibodies, including goat anti‐rabbit HRP (FDR007, 1:5000, Fudebio‐tech) and goat anti‐mouse HRP (FDM007, 1:5000, Fudebio‐tech), for 2 h at room temperature. Target protein bands were detected by enhanced chemiluminescence (P10300, NCM Biotech) and visualized with the ChemiDoc XRS+ imaging system (Bio‐Rad, Hercules, CA, USA). Band intensities were semi‐quantified using ImageJ software (US National Institutes of Health, Bethesda, MD, USA). Results were expressed as the ratio of target protein intensity to the corresponding internal loading control.

### 4.9 IF Staining In Vivo

Mice were deeply anesthetized and transcardially perfused with ice‐cold 1× phosphate‐buffered saline (PBS) followed by 1× paraformaldehyde (PFA). Brains were rapidly removed and post‐fixed in 1× PFA overnight at 4°C. Tissues were then cryoprotected in 30% sucrose at 4°C for 48–72 h and sectioned into 8‐ or 20‐µm slices using a cryostat microtome (8 µm for FerroOrange staining, 20 µm for all other IF staining). Sections were dried for 30 min at 37°C and stored at −20°C until use. The sections were first blocked with 2% bovine serum albumin (BSA) in PBS + 1.25% Triton X‐100 for 1 h, washed three times with 1× PBS for 5 min each, and incubated overnight at 4°C with the following primary antibodies: rabbit anti‐MBP (OB‐PRB130‐01, 1:500, Oasis, Zhejiang, China), rat anti‐CC1 (OB‐PRT039‐02, 1:300, Oasis), Filipin (SAE0088, 1:2, Sigma), BODIPY 493/503 (121207‐31‐6, Amgicam), rabbit anti‐CASPR (55417‐1‐AP, 1:300, Proteintech, Rosemont, IL, USA), mouse anti‐NRAMP1 (sc‐398077, 1:100, Santa Cruz, Dallas, TX, USA), FluoroMyelin (F34652, 1:300, Thermo Fisher Scientific), guinea pig anti‐IBA1 (OB‐PGP049‐02, 1:500, Oasis), mouse anti‐LAMP1 (sc‐20011, 1:300, Santa Cruz), FerroOrange (F374, 1:500, DOJINDO, Kumamoto, Japan), rabbit anti‐ABCA1 (ab52617, 1:300, Abcam), rabbit anti‐CYP46A1 (12486‐1‐AP, 1:300, Proteintech), rabbit anti‐LXRβ (ab315082, 1:300, Abcam), rabbit anti‐dMBP (AB5864, 1:300, Abcam), rat anti‐CD68 (ab53444, 1:300, Abcam), rabbit anti‐ABCG1 (NB400‐105SS, 1:300, Novus)rabbit anti‐TFEB (13372‐1‐AP, 1:500, Proteintech), rabbit anti‐FTL (ab109373, 1:500, Abcam), and rabbit anti‐FTH (ab183781, 1:500, Abcam). After incubation, sections were washed three times with 1× PBS for 5 min each at room temperature. Sections were then incubated for 2 h at room temperature in the dark with secondary antibodies and subsequently mounted on coverslips with 4′,6‐diamidino‐2‐phenylindole (DAPI; 0100–20, SouthernBiotech, Birmingham, AL, USA) to stain the nuclei. Secondary antibodies included goat anti‐rabbit IgG (H+L), Alexa Fluor 488 (AB0141, 1:500, Abways, Shanghai, China), CoraLite488‐conjugated AffiniPure goat anti‐mouse IgG (H+L) (SA00013‐1, 1:500, Proteintech), CoraLite594‐conjugated goat anti‐mouse IgG (H+L) (SA00013‐3, 1:500, Proteintech), CoraLite594‐conjugated goat anti‐rabbit IgG (H+L) (SA00013‐4, 1:500, Proteintech), CoraLite647‐conjugated AffiniPure F(ab′)2 fragment goat anti‐mouse IgG (H+L) (SA00014‐10, 1:500, Proteintech), goat anti‐guinea pig IgG (H+L), 488 nm (G‐GP488, 1:500, Oasis), goat anti‐guinea pig IgG, 647 nm (OB‐GP647, 1:500, Oasis), and donkey anti‐rat IgG (H+L), highly cross‐adsorbed secondary antibody, Alexa Fluor 488 (A‐21208, 1:500, Thermo Fisher Scientific). Images were obtained using a confocal microscope (LSM 800, Zeiss, Oberkochen, Germany).

### 4.10 qPCR

Total RNA of brain tissues was isolated using TRIzol reagent according to the manufacturer's protocol. Total RNA was extracted with TIANGEN RNApre Pure Tissue kit. GAPDH was used as the internal control. The sequences of qRT‐PCR primers for the genes examined are listed below: mouse ABCA1 forward primer: 5’‐GGGTGTCTACGTGCAACAGA‐3’, reverse primer: 5’‐GCGACAGAGTAGATCCAGGC‐3’; mouse ABCG1 forward primer: 5’‐TTTTGTGCTGTTCGCTGCTC‐3’, reverse primer: 5’‐CACAAATGTCGCAACCTGCA‐3’; mouse LXRβ forward primer: 5’‐CCCCACCCATTGAGTCTTCC‐3’, reverse primer: 5’‐CTCCACTCAAGGTGCATGGT‐3’; mouse CYP46A1 forward primer: 5’‐AAGAGGGAGCTCAGGACGAT‐3’, reverse primer: 5’‐AACCGACAACCTCATCCACC‐3’.

### 4.11 Bulk RNA‐Seq Analysis

Mice from the L‐NIO and sham‐operated groups were sacrificed on day 7 post‐modeling. Brains were rapidly removed, and lesions were dissected from the corpus callosum on ice. Samples from both groups were submitted to GENEWIZ Technology Co. (Suzhou, China) for total RNA extraction, bulk RNA‐seq library construction, and sequencing. Raw sequencing data were processed using differential expression analysis for sequence count data 2. All statistical analyses were performed in R version 4.3.1 (The R Foundation for Statistical Computing, Vienna, Austria). Genes were considered statistically significant if they met the criteria of *p* < 0.05 and |log_2_ (fold change)| ≥ 1.0.

### 4.12 Preparation of Cells for scRNA‐Seq

Two weeks after the injection of *Slc11a1*‐shRNA or NC‐shRNA lentivirus into the corpus callosum of C57BL/6 mice, L‐NIO was stereotactically injected into the same site. Mice were sacrificed after L‐NIO administration, and corpus callosum lesions were rapidly dissected on ice. Dissected tissues from both groups were submitted to CapitalBio Technology Co. (Beijing, China) for RNA extraction, library construction, and sequencing. Tissues were minced into small fragments, transferred into centrifuge tubes, and incubated with enzymatic digestion buffer. The resulting supernatant was treated with red blood cell lysis buffer and incubated for 5 min at room temperature. The mixture was then centrifuged at 400 × *g* for 5 min at 4°C, and the pellet was resuspended in PBS.

### 4.13 Cell Capture and cDNA Synthesis

Cell suspensions were loaded onto a Chromium Single Cell Controller (10x Genomics, Pleasanton, CA, USA) to generate Gel Bead‐in‐Emulsion (GEMs) using the Chromium Single Cell A Chip Kit (120236, 10x Genomics) and Gel Bead Kit v2 (120237, 10x Genomics) in accordance with the manufacturer's instructions. GEMs were reverse‐transcribed under the following conditions: 53°C for 45 min, 85°C for 5 min, and held at 4°C. The resulting single‐stranded cDNA was purified and subsequently amplified.

### 4.14 ScRNA‐seq Library Preparation

ScRNA‐seq libraries were prepared using the Single Cell 3′ Library & Gel Bead Kit v2 (1120237, 10x Genomics) in accordance with the manufacturer's instructions.

### 4.15 ScRNA‐seq

ScRNA‐seq was performed on an Illumina NovaSeq 6000 platform. Raw sequencing data in FASTQ format were processed using Cell Ranger software (version 3.0, 10x Genomics). The “cellranger count” function was used to align the original sequencing reads to the mouse reference genome and quantify gene expression by counting barcodes and unique molecular identifiers in each cell. ScRNA‐seq data analysis was performed using R version 4.3.1 and the Seurat package (version 4.4.0). Cells and genes were excluded using the following quality control criteria: expression in fewer than three cells, fewer than 1000 or more than 5000 detected genes per cell, and mitochondrial gene content exceeding 5%. After filtering and quality control, high‐quality cells were retained for downstream bioinformatic analyses. The raw count matrix was normalized using the Seurat “LogNormalize” method, which normalizes gene expression by the total expression per cell and multiplies by a scale factor of 10,000. Highly variable genes were identified using the “FindVariableFeatures” function (method = “vst,” 2000 features per dataset). Principal component analysis was then performed on the top 2000 highly variable genes to reduce dimensionality. The top 20 principal components were selected for further analysis, as determined using Seurat's “ElbowPlot” and “JackStrawPlot” functions. The Harmony package was utilized to correct for batch effects between groups. Clustering analysis was performed using Seurat's “FindNeighbors” and “FindClusters” functions with a resolution parameter of 0.6. For dimensionality reduction and visualization, UMAP and t‐SNE were used to construct 2D representations of cell similarity. The “FindAllMarkers” function in Seurat was used to identify marker genes for each cluster. Cell type annotation was performed by comparing the gene expression profiles and the top 10 marker genes of each cluster to reference markers in the CellMarker 2.0 database (http://www.bio‐bigdata.center/) and previously published literature [35, 67, 68, 69, 70, 71, 72]. In total, 11 cell clusters were annotated. Because microglia constituted the majority of the captured cells and SLC11A1 was primarily expressed in microglia, the same Seurat workflow and downstream subclustering analyses were applied specifically to the microglial population.

### 4.16 Gene Enrichment and Pathway Analysis

GSVA was performed on the expression matrix of microglial subclusters using the “GSVA” and “msigdbr” R packages. Gene sets were specified for *Mus musculus* with category “C5.” GSVA results were visualized as heatmaps. DEGs were defined using the criteria of absolute log_2_ fold change >0.8 and adjusted *p*‐value <0.05 when comparing cluster 5 with other microglial clusters. GO analysis, KEGG analysis, and GSEA were conducted on DEGs and key marker genes distinguishing cluster 5 from other clusters using the “ClusterProfiler” package. The analysis focused on differences between the *Slc11a1*‐shRNA and NC‐shRNA groups. DEGs were identified using adjusted *p*‐value <0.05 when comparing the two groups within cluster 5. Functional enrichment analyses, including GO, KEGG, and GSEA, were performed to interpret biological significance. GSVA was also applied to raw data from microglia and cluster 5.

### 4.17 EdU Injection and Staining

Two weeks after stereotactic injections of DIO‐*Slc11a1*‐shRNA or DIO‐NC‐shRNA LV into *Cx3cr1* ^CreERT2^ mice followed by tamoxifen administration, L‐NIO was delivered to both groups. To label newly generated cells in vivo, mice received intraperitoneal injections of EdU (5 mg kg^−1^, BD103898, Bidepharm, Shanghai, China) every 12 h from day 4 to day 14 post‐stroke (11 consecutive days). EdU staining was performed using the Click‐iT EdU Imaging Kit according to the manufacturer's protocol (C0081S, Beyotime).

### 4.18 Molecule Dynamics

All‐atom molecular dynamics (MD) simulations were performed using the docked protein–ligand complexes as the starting structures. The Y484A mutant was constructed from the wild‐type protein with PyMOL 2.5.5. MD simulations were carried out with AMBER 24. Prior to the simulations, the ligand charges were derived with the antechamber module based on Hartree–Fock (HF) SCF/6‐31G* calculations using Gaussian 09. The ligand and protein were parameterized with the GAFF2 small‐molecule force field and the ff14SB protein force field, respectively. Hydrogen atoms were added with the LEaP module, and each system was solvated in a truncated octahedral TIP3P box extending 10 Å from the solute. Na^+^ and Cl^−^ ions were added to neutralize the system, and the resulting topology and parameter files were generated for simulation. Energy minimization was performed with 2500 steps of steepest descent followed by 2500 steps of conjugate gradient. The system was then gradually heated from 0 to 298.15 K over 200 ps at constant volume. This was followed by 500 ps of NVT equilibration at 298.15 K to allow uniform solvent distribution, and then 500 ps of NPT equilibration at 1 atm. Production MD was run for 100 ns under NPT conditions with periodic boundary conditions. During production, a 10 Å cutoff was applied for nonbonded interactions. Long‐range electrostatics were treated with the particle mesh Ewald (PME) method, and all bonds involving hydrogen were constrained using the SHAKE algorithm. Temperature was controlled by a Langevin thermostat with a collision frequency (γ) of 2 ps^−^^1^, and a 2 fs integration step was used. Trajectories were saved every 10 ps for subsequent analysis. The binding free energies between the protein and ligand in all systems were calculated using the MM/GBSA method. In this study, the MD trajectories from 90–100 ns were used for the calculations.

### 4.19 Myelin Preparation

Myelin was isolated from the brains of six adult C57BL/6 mice according to a previously published protocol [73]. Briefly, adult mice were anesthetized and perfused with ice‐cold 1× PBS to remove blood. Brains were promptly harvested and minced on ice. Myelin was extracted using sucrose density gradient centrifugation, repeatedly washed, and subjected to osmotic shock via ultracentrifugation (Beckman Coulter, Brea, CA, USA) at 4°C until a highly enriched fraction was obtained. The purified myelin was resuspended in 200 µL of cold 1× Tris‐buffered saline and stored at −80°C.

### 4.20 In Vitro Grouping

For all *Slc11a1* knockdown experiments in vitro, primary microglia were assigned to one of four groups, namely *Slc11a1*‐shRNA with or without myelin and NC‐shRNA with or without myelin.

### 4.21 Primary Microglia Isolation, Culture, and Treatment

Primary microglia were isolated from the cerebral cortices of postnatal day 0–3 C57BL/6 mice as previously described [74]. Briefly, neonatal brains were digested with trypsin and dissociated into single‐cell suspensions. Cells were plated onto poly‐d‐lysine–coated Nunc EasYFlasks (P6407‐5MG, Sigma‐Aldrich) and maintained in complete culture medium [Dulbecco's modified Eagle medium (DMEM)/F‐12 supplemented with 10% fetal bovine serum (FBS) and 1% penicillin–streptomycin) at 37°C under 5% CO_2_. Culture medium was refreshed every 3 days. On day 14, flasks were shaken at 37°C for 30 min. Detached cells were collected by centrifugation at 1500 rpm for 5 min, resuspended in fresh complete medium, and seeded on round glass coverslips in 24‐well plates according to the group designation. Cells were used for further analysis 24 h after seeding.

### 4.22 BV2 Cell Culture and Treatment

BV2 cells were cultured in complete culture medium (DMEM supplemented with 10% FBS and 1% penicillin–streptomycin) at 37°C under 5% CO_2_. Cells were seeded on round glass coverslips in 24‐well plates according to the group designation.

### 4.23 In Vitro Lentiviral Infection

Primary microglia were infected with either *Slc11a1*‐shRNA or NC‐shRNA lentivirus at the indicated concentrations for 48 h prior to downstream analyses.

### 4.24 Myelin Phagocytosis and Degradation in Vitro

Myelin was labeled with PKH26 (MINI26‐1KT, Sigma–Aldrich) according to the manufacturer's protocol. Briefly, myelin (1 µg mL^−1^) was mixed with 0.4 µL of PKH26 ethanolic dye solution and 100 µL of Diluent C (Sigma–Aldrich). The mixture was incubated in the dark for 5 min with intermittent shaking to ensure uniform staining. To terminate the labeling reaction, 100 µL of FBS was added, followed by incubation for 1 min. The mixture was centrifuged at 7500 rpm for 10 min at 20–25°C. The myelin pellet was washed three times with 200 µL of 1× PBS, with centrifugation after each wash. All steps were performed in the dark. After the final wash, the supernatant was discarded, and the pellet was resuspended in 1000 µL of complete culture medium (DMEM/F‐12 supplemented with 10% FBS and 1% penicillin–streptomycin). For the myelin phagocytosis assay, *Slc11a1*‐shRNA–or control lentivirus‐infected primary microglia were cultured in complete medium with or without labeled myelin and incubated for 3.5 h at 37°C in a humidified incubator. For the degradation assay, cells were incubated with or without labeled myelin for 6 h before IF staining.

### 4.25 IF Staining In Vitro

After incubation, the culture medium was removed from all four groups. Cells were washed twice with 1× PBS for 5 min each and then fixed with 1× PFA for 20 min at room temperature. Fixed cells were subsequently washed three times with 1× PBS for 5 min each. Cell membranes were permeabilized via incubation with 0.2% Triton X‐100 for 10 min at room temperature, followed by another PBS wash for 5 min. Cells were blocked with 5% BSA prepared in 10% FBS (diluted in 1× PBS) for 1 h at 37°C and then washed with PBS. Next, cells were incubated overnight at 4°C with mouse anti‐LAMP1, guinea pig anti‐IBA1, and rabbit anti‐TFEB antibodies. After two washes with PBS, cells were incubated with secondary antibodies, namely CoraLite647‐conjugated AffiniPure F(ab′)2 fragment goat anti‐mouse IgG (H+L), CoraLite594‐conjugated goat anti‐rabbit IgG (H+L), and goat anti‐guinea pig IgG, 647 nm, for 2 h at room temperature. Coverslips were mounted with DAPI‐containing mounting medium and imaged using a confocal microscope.

### 4.26 TFEB Staining In Vitro

Primary microglia infected with *Slc11a1*‐shRNA or NC‐shRNA lentivirus were incubated in complete culture medium with or without unlabeled myelin for 3 h at 37°C in a cell culture incubator. After incubation, IF staining was performed as previously described.

### 4.27 Live‐Cell Imaging

Lentivirus‐infected cells were treated with or without unlabeled myelin for 2 h at 37°C in an incubator. For co‐treatment assays, cells were incubated with myelin and LM22B‐10 for 2 h prior to imaging. To assess intracellular Fe^2+^ levels [26], cells were stained with FerroOrange (F374, 1:700, DOJINDO), LysoTracker Deep Red (L12492, 50 nM, Thermo Fisher Scientific), and Hoechst 33342 for 30 min at 37°C. After staining, cells were immediately transferred to a confocal microscope for imaging under constant acquisition parameters. To evaluate lysosomal pH, cells were stained with LysoSensor Yellow/Blue DND‐160 (L7545, 5 µM, Thermo Fisher Scientific) for 5 min at 37°C as previously reported [75]. After staining, cells were washed with PBS to remove excess dye and promptly transferred to the confocal microscope. Excitation was performed at (blue) and 384 nm (yellow), and emission was captured at (blue) and 540 nm (yellow). Fluorescence at 440 nm indicated less acidic or impaired lysosomes, whereas that at 540 nm indicated more acidic, functional lysosomes. The results were expressed as the I_540_/I_440_ ratio per cell.

### 4.28 Virtual Screening for Small‐molecule Compounds Targeting Mouse SLC11A1

The structure of the mouse SLC11A1 protein was obtained from the AlphaFold database (AlphaFold ID: AF‐P41251‐F1). High‐throughput virtual screening was conducted by MedChemExpress to identify potential specific antagonists of SLC11A1. Compounds from two commercial libraries were screened using high‐throughput virtual screening, standard precision, and extra precision docking protocols in Schrödinger Maestro software (version 11.4). The top 200 small‐molecule candidates were selected according to molecular docking scores.

### 4.29 Statistical Analysis

GraphPad Prism 9 software and RStudio (version 4.3.1) were used for statistical analysis. Data normality was assessed by the Shapiro–Wilk test. One‐way analysis of variance (ANOVA) was applied for multiple‐group comparisons, and the two‐tailed unpaired Student's *t*‐test was used for two‐group comparisons. Data were expressed as the mean ± SEM. *p* < 0.05 was considered statistically significant.

## Author Contributions

L.Q. performed most of the experiments, analyzed the data, and drafted the manuscript. Y.C., J.C., and J.J. contributed to conceptualization, study design, and data interpretation. C.L. interpreted data. Y. Z., Y.T., H.H., J.X., Y.Z., H.W., and Y.W. contributed to part of the animal experiments, primary microglia culture, immunostaining, mice genotyping and WB. All authors have read and approved the final manuscript.

## Conflicts of Interest

The authors declare no conflicts of interest.

## Supporting information




**Supporting File**: advs74040‐sup‐0001‐SuppMat.docx.

## Data Availability

The data that support the findings of this study are available from the corresponding author upon reasonable request.
