# Supplementary material for: Inhibition of SLC11A1‐Mediated Lysosomal Iron Accumulation in Microglia Promotes Repair Following White Matter Stroke
Source: Adv Sci (Weinh). 2026 Jan 25;13(19):e11482. doi: 10.1002/advs.202511482 (PMC13045239; doi:10.1002/advs.202511482)
Supplement: Supplementary file 1 — Supporting File: advs74040‐sup‐0001‐SuppMat.docx. [file ADVS-13-e11482-s001.docx]

Supporting Information

**Inhibition of SLC11A1-Mediated Lysosomal Iron Accumulation in Microglia Promotes Repair Following White Matter Stroke**

Lingling Qiu | Yajie Zhang | Yushi Tang | Hongli Hu | Ying Zhang | Junwen Xue | Hao Wang | [Yecheng Wang](https://pubmed.ncbi.nlm.nih.gov/?sort=pubdate&term=Wang+Y&cauthor_id=39228046) | Chunfeng Liu | Jia Jia* | Jian Cheng* | Yongjun Cao*

**This file includes:**

Supplementary Figure S1-S17

**Figure S1.** The lysosomal pathway and SLC11A1 are involved in the pathophysiological process of ischemic white matter injury. A) Myelin basic protein (MBP) immunofluorescence staining showing representative images of infarct lesions on day 4, week 2, and week 9 after modeling (n = 3). White arrows indicate lesion sites. Scale bar 500 μm. B) Bar chart visualizing gene set enrichment analysis (GSEA) results. Ipsilateral, ips. C) GSEA results indicate substantial activation of the lysosomal pathway. D, E) Representative images showing co-labeling of SLC11A1 (red) and ionized calcium-binding adaptor molecule 1 (IBA1) (green) in the sham and L-NIO groups on days 4, 7, and 14 post-stroke (D; scale bar 20 μm), and corresponding quantification data (E; each dot represents the average of three fields per brain section, n = 3 sections per mouse, N = 4 mice per group; welch’s ANOVA test followed by Dunnett's T3 post hoc test; 4d L-NIO vs 7d L-NIO: 95% *CI*[ -0.6104, 0.08996]). F, G) Immunoblot bands showing SLC11A1 expression levels in the sham and L-NIO groups at various time points post-stroke (F), and corresponding quantification data (G; N = 5 ; one-way ANOVA followed by Tukey's post hoc test; 4d L-NIO vs 7d L-NIO: 95% *CI*[-0.5104 0.03601]). *β*-actin served as an internal control. H, I) Immunoblot bands showing SLC11A1 expression on day 7 post-modeling (H), and corresponding quantification data (I; N = 6; one-way ANOVA followed by Tukey's post hoc test; L-NIO Ipsil vs LPC Ipsil: 95% *CI*[0.1373, 0.3527]). *β*-actin served as an internal control. Ipsilateral, Ipsil; contralateral, Contra. Data are presented as the mean ± SEM. **p* < 0.05, ***p* < 0.01, ****p* < 0.001, *****p* < 0.0001, ns, no significance.

**Figure S2.** Iron and myelin debris are deposited in activated microglial lysosomes within white matter injury lesions. A, B) Representative images showing 4',6-diamidino-2-phenylindole (DAPI; blue), IBA1 (green), FluoroMyelin (red), and LAMP1 (purple) at week 3 post-stroke (A; white arrows indicate colocalization of FluoroMyelin with LAMP1, IBA1; scale bar 20 μm), and corresponding quantification data (B; each dot represents the average of three fields per brain section, n = 3 sections per mouse, N = 3 mice per group; two-tailed unpaired Student’s *t*-test with Welch's correction; 95% *CI*[-20146, -16879]). C, D) Representative images showing DAPI (blue), IBA1 (green), FluoroMyelin (red), and LAMP1 (purple) at week 9 post-stroke (C; white arrows indicate colocalization of FluoroMyelin with LAMP1, IBA1; scale bar 20 μm), and corresponding quantification data (D; each dot represents the average of three fields per brain section, n = 3 sections per mouse, N = 4 mice per group; two-tailed unpaired Student’s *t*-test with Welch's correction; 95% *CI*[-26572, -14684]). E) Representative images of Caspr immunofluorescence staining at week 9 post-stroke (three fields per brain section, n = 3 sections per mouse, N = 5 mice per group; scale bar 10 μm). F-M) Immunoblot bands showing expression levels of FTH, FTL, FPN, TFR1, 4-HNE, ACSL4, and GPX4 in sham and L-NIO groups at various time points post-stroke (F), and corresponding quantification data (G-M; FTH and FTL, N = 6; FPN, TFR1, 4-HNE, ACSL4, and GPX4, N = 5; one-way ANOVA followed by Tukey's post hoc test;4d L-NIO vs 7d L-NIO: FTH 95% *CI*[-0.6724, 0.093546], FTL 95% *CI*[-0.1992, 0.5526], 4-HNE 95% *CI*[-0.5684, 0.1659]; 2d L-NIO vs 4d L-NIO: TFR1 95% *CI*[-0.5502, -0.01594], ACSL4 95% *CI*[-0.4210, -0.02422], GPX4 95% *CI*[-0.1333, 0.6717]; 24h L-NIO vs 2d L-NIO: FPN 95% *CI*[-0.3463, -0.04772]). *β*-actin served as an internal control. Data are presented as the mean ± SEM. **p* < 0.05, ***p* < 0.01, ****p* < 0.001, *****p* < 0.0001, ns, no significance.

**Figure S3.** Iron is deposited in activated microglial lysosomes within white matter injury lesions. A-F) Representative images showing DAPI (blue), IBA1 (green), FerroOrange (red, Fe^2+^), FTH (red, Fe^3+^), FTL (red, Fe^3+^), and LAMP1 (purple) on day 7 post-stroke in the L-NIO and LPC groups (A, C, E; scale bar 20 μm), and corresponding quantification data (B, D, F; each dot represents the average of three fields per brain section, n = 3 sections per mouse, N = 5 mice per group; two-tailed unpaired Student’s *t*-test with Welch's correction; FerroOrange 95% *CI*[-0.5351, -0.3699], FTH 95% *CI*[-0.4659, -0.3295], FTL 95% *CI*[-0.3842, -0.2606]). G-N) Immunoblot bands showing expression levels of FTH, FTL, FPN, TFR1, 4-HNE, ACSL4, and GPX4 in the L-NIO and LPC groups (G; FTH, FTL, and 4-HNE on day 7 post-stroke; ACSL4, TFR1, and GPX4 on day 4; FPN on day 2), and corresponding quantification data (H-N; GPX4, N = 6; all others, N = 5; one-way ANOVA followed by Tukey's post hoc test; L-NIO Ipsil vs LPC Ipsil: FTH 95% *CI*[0.4267, 0.7847], FTL 95% *CI*[0.3067, 0.7715], FPN 95% *CI*[0.08034, 0.3537], TFR1 95% *CI*[0.2340, 0.6328], 4-HNE 95% *CI*[0.3194, 0.5470], ACSL4 95% *CI*[0.1897, 0.5201], GPX4 95% *CI*[-1.295, -0.2827]). *β*-actin served as an internal control. Data are presented as the mean ± SEM. ***p* < 0.01, *****p* < 0.0001, ns, no significance.

**Figure S4.** Iron chelation attenuates iron accumulation. A-D) Representative images of DAPI (blue), IBA1 (green), FTH (red, Fe^3+^), FTL (red, Fe^3+^), and LAMP1 (purple) on day 7 post-stroke (A, C; scale bar 20 μm) in the DFO and control groups, with corresponding quantification data (B, D; each dot represents the average of three fields per brain section, n = 3 brain sections per mouse, N = 5 mice per group; B; two-tailed unpaired Student’s *t*-test; D; two-tailed unpaired Student’s *t*-test with Welch's correction; FTH 95% *CI*[-0.1841, -0.1409], FTL 95% *CI*[-0.4591, -0.3304]). E-L) Representative immunoblot images showing expression levels of FTH, FTL, FPN, TFR1, 4-HNE, ACSL4, and GPX4 in the DFO and control groups (E; FTH, FTL, and 4-HNE on day 7 post-stroke; ACSL4, TFR1, FPN, and GPX4 on day 4), with corresponding quantification data (F to L; FTH, N = 4; all others, N = 5; one-way ANOVA followed by Tukey's post hoc test; Veh+L-NIO Ipsil vs DFO+L-NIO Ipsil: FTH 95% *CI*[0.1039, 0.9535], FTL 95% *CI*[0.07144, 0.7170], FPN 95% *CI*[-0.2491, -0.09661], TFR1 95% *CI*[0.2130, 0.8564], 4-HNE 95% *CI*[0.001087, 0.5593], ACSL4 95% *CI*[0.01919, 0.5189], GPX4 95% *CI*[-0.5473, -0.1729]). *β*-actin served as an internal control. Data are presented as the mean ± SEM. **p* < 0.05, ***p* < 0.01, ****p* < 0.001, *****p* < 0.0001, ns, no significance.

**Figure S5.** SLC11A1 knockdown promotes white matter recovery. A, B) Immunoblot showing SLC11A1 knockdown efficiency in different groups (A) and corresponding quantification (B; N = 5; one-way ANOVA followed by Tukey's post hoc test; *Slc11a1*-shRNA Ipsil vs Contra:95% *CI*[-0.3331, -0.05523]). *β*-actin served as an internal control. C) IF showing SLC11A1 knockdown efficiency. Scale bar 200 μm. D, E) Representative images of infarct lesions at week 9 post-stroke in *Slc11a1*-shRNA–treated and control groups (D; scale bar 500 μm), and corresponding quantification (E; N = 7 per group; two-tailed unpaired Student’s *t*-test; 95% *CI*[-0.004636, -0.003017]). White arrows indicate lesion sites. F) IF showing Caspr expression in various groups (three fields per brain section, n = 3 brain sections per mouse, N = 5 mice per group; scale bar 10 μm). G, H) Novel object recognition (G) and grid walking tests (H) at weeks 2, 4, and 9 post-stroke in sham, *Slc11a1*-shRNA–treated, and control groups (N = 10 per group; one-way ANOVA followed by Tukey's post hoc test; G *Slc11a1*-shRNA vs NC-shRNA: Week 2 95% *CI*[-25.16, 6.494], Week 4 95% *CI*[-36.20, -12.80], Week 9 95% *CI*[-28.96, -5.851]; H: *Slc11a1*-shRNA vs NC-shRNA:Week 9 95% *CI*[0.7893, 5.065]). I-L) Representative images of DAPI (blue), IBA1 (green), FluoroMyelin (red), and LAMP1 (purple) at week 3 (I) and week 9 (K) post-stroke(I, K; white arrows indicate colocalization of FluoroMyelin with LAMP1, IBA1; scale bar 20 μm), with corresponding quantification (J, L; each dot represents the average of three fields per brain section, n = 3 brain sections per mouse; J: N = 4 mice per group, L: N = 5 mice per group; two-tailed unpaired Student’s *t*-test with Welch's correction; Week 3 95% *CI*[14647, 42214], Week 9 95% *CI*[-33764, -16755]). M, N) Representative images of DAPI (blue), IBA1 (green), FerroOrange (red), and LAMP1 (purple) on day 7 post-stroke (M; scale bar 20 μm), and statistical analysis (N; each dot represents the average of three fields per brain section, n = 3 brain sections per mouse, N = 5 mice per group; two-tailed unpaired Student’s *t*-test with Welch's correction; 95% *CI*[-0.2140, -0.07275]). Data are presented as the mean ± SEM. ***p* < 0.01, ****p* < 0.001, *****p* < 0.0001, ns, no significance.

**Figure S6.** Specific knockdown of SLC11A1 in *Cx3cr1*^CreERT2^ mice. A) Schematic showing experimental design for microglia-specific *Slc11a1* knockdown*.* B) Timeline of the experimental procedure. C) Representative images confirming microglia-

specific targeting, N = 3, scale bar 20 μm. D, E) Immunoblot analysis of SLC11A1 knockdown efficiency in *Cx3cr1*^CreERT2^ mice (D) and corresponding quantification (E; N = 5; one-way ANOVA followed by Tukey's post hoc test; Ipsil vs Contra:95% *CI*[-0.4708, -0.06113]). *β*-actin served as an internal control. F) qPCR for *Abca1* (N = 6), *Abcg1* (N = 6), *Lxrβ* (N = 6)*, and Cyp46a1* (N = 5) mRNA expression in the *Slc11a1*-shRNA-treated and NC-shRNA-treated groups on days 7 post-stroke (welch’s ANOVA test followed by Dunnett's T3 post hoc test; *Slc11a1*-shRNA vs NC-shRNA: *Abca1* 95% *CI*[0.4601, 0.8901], *Abcg1* 95% *CI*[0.6290, 1.040], *Lxrβ* 95% *CI*[0.05420, 0.4570], *Cyp46a1* 95% *CI*[1.193, 2.113]). G, H) Representative images showing co-labeling of IBA1 (green), FerrOrange (red), and CD68 (purple, an activated microglia marker) on days 3 and 7 post-stroke (G; scale bar 20 μm), and corresponding quantification data (H; each dot represents a field, three fields per brain section, n = 3 sections per mouse, N = 3 mice per group; two way ANOVA test followed by Tukey's test; 3d Ipsil vs 7d Ipsil: 95% *CI*[-0.8558, -0.5025]). Data are presented as the mean ± SEM. **p* < 0.05, ***p* < 0.01, ****p* < 0.001, *****p* < 0.0001, ns, no significance..

**Figure S7.** SLC11A1 overexpression impedes white matter recovery in a repairable LPC-induced demyelination model. A, B) Representative immunoblot images showing SLC11A1 overexpression efficiency (A) and corresponding statistical analysis (B; N = 5; one-way ANOVA followed by Tukey's post hoc test; LV-*Slc11a1* Ipsil vs Contra:95% *CI*[0.01415, 0.5733]). *β*-actin served as an internal control. C, D) MBP staining showing infarct lesions in lentivirus-*Slc11a1-*treated and control mice (C; scale bar 500 μm), with quantification (D; N = 7 per group; two-tailed unpaired Student’s *t*-test with Welch's correction; 95% *CI*[0.006377, 0.007363]). White arrows indicate infarct lesions. E) IF showing Caspr expression (three fields per brain section, n = 3 brain sections per mouse, N = 5 mice per group; scale bar 10 μm). F, G) Assessment of neurological deficits using novel object recognition (F) and grid walking tests (G) at weeks 2, 4, and 9 after LPC modeling (N = 10 per group; one-way ANOVA followed by Tukey's post hoc test; F *Slc11a1*-shRNA vs NC-shRNA: Week 2 95% *CI*[1.347, 29.89], Week 4 95% *CI*[0.8752, 34.97], Week 9 95% *CI*[3.475, 38.01]; G: LV-*Slc11a1* vs LV-NC:Week 9 95% *CI*[-2.932, -1.623]). H-K) Representative images of DAPI (blue), IBA1 (green), FluoroMyelin (red), and LAMP1 (purple) at week 3 (H) and week 9 (J) post-stroke(H,J; scale bar 20 μm; white arrows indicate colocalization of FluoroMyelin with LAMP1, IBA1), with corresponding quantification (I, K; each dot represents the average of three fields per brain section, n = 3 brain sections per mouse; I: N = 4 mice per group; K: N = 5 mice per group; two-tailed unpaired Student’s *t*-test with Welch's correction; Week 3 95% *CI*[23860, 34655], Week 9 95% *CI*[26433, 51811]). L, M)Representative images of DAPI (blue), IBA1 (green), FerroOrange (red, Fe^2+^), and LAMP1 (purple) on day 7 after LPC modeling (L; scale bar 20 μm) in LV-*Slc11a1*–treated and control groups, with corresponding quantification (M; each dot represents the average of three fields per brain section, n = 3 brain sections per mouse, N = 5 mice per group; two-tailed unpaired Student’s *t*-test with Welch's correction; FerroOrange 95% *CI*[0.1004, 0.2031]). Data are presented as the mean ± SEM. **p* < 0.05, ***p* < 0.01, ****p* < 0.001, *****p* < 0.0001, ns, no significance.

**Figure S8.** SLC11A1 overexpression increases iron accumulation. A-D) Representative images of DAPI (blue), IBA1 (green), FTH (red, Fe^3+^), FTL (red, Fe^3+^), and LAMP1 (purple) on day 7 after LPC modeling (A, C; scale bar 20 μm) in LV-*Slc11a1*–treated and control groups, with corresponding quantification (B, D; each dot represents the average of three fields per brain section, n = 3 brain sections per mouse, N = 5 mice per group; two-tailed unpaired Student’s *t*-test with Welch's correction; FTH 95% *CI*[0.2240, 0.4532], FTL 95% *CI*[0.1829, 0.2655]). E-L) Representative immunoblot images showing expression levels of FTH, FTL, FPN, TFR1, 4-HNE, ACSL4, and GPX4 in LV-*Slc11a1*–treated and control groups (E; FTH, FTL, and 4-HNE assessed on day 7 post-stroke; ACSL4, TFR1, and GPX4 on day 4; FPN on day 2), with corresponding quantification (F-L; N = 5; one-way ANOVA followed by Tukey's post hoc test; LV-NC vs LV-*Slc11a1:* FTH 95% *CI*[-1.198, -0.5579], FTL 95% *CI*[-0.8532, -0.5086], FPN 95% *CI*[0.03185, 0.2797], TFR1 95% *CI*[-0.7912, -0.4816], 4-HNE 95% *CI*[-0.5601, -0.1925], ACSL4 95% *CI*[-0.4619, -0.2443], GPX4 95% *CI*[0.3671, 1.128]). *β*-actin served as an internal control. Data are presented as the mean ± SEM. **p* < 0.05, ****p* < 0.001, *****p* < 0.0001, ns, no significance.

**Figure S9.** ScRNA-seq reveals cell type proportion of SLC11A1 knockdown. A, B) Pie chart showing the proportion of 11 clusters in *Slc11a1*-shRNA-L-NIO group and NC-shRNA-L-NIO group. C) t-distributed stochastic neighbor embedding (t-SNE) plot of macrophage subdivided into 5 clusters. D) uniform manifold approximation and projection (UMAP) plot of macrophage subdivided into 5 clusters. E) Dot plot displaying selected DEGs for each cluster. F) Violin plot showing SLC11A1 expression across various macrophage subclusters (Subcluster 0: avg_log2FC = -0.170, adjusted *P* = 0.140; subcluster 1: avg_log2FC = -0.164, adjusted *P* = 1.0; subcluster 2: avg_log2FC = 0.070, adjusted *P* = 1.0; subcluster 3: avg_log2FC = 0.167, adjusted *P* = 1.0; subcluster 4: avg_log2FC = -0.261, adjusted *P* = 1.0). G) UMAP plot of microglia subdivided into 8 clusters.

**Figure S10.** Specific knockdown of CTSD in *Cx3cr1*^CreERT2^ mice impedes white matter recovery in a repairable LPC-induced demyelination model. A, B) CTSD knockdown efficiency in *Cx3cr1*^CreERT2^ mice (A), and corresponding quantification (B; N = 5 per group; one-way ANOVA followed by Tukey's post hoc test; *DIO-Ctsd*-shRNA Ipsil vs Contra:95% *CI*[0.1005, 0.5298]). *β*-tubulin served as an internal control. C, D) Representative images of MBP staining showing infarct lesions in DIO-*Ctsd*-shRNA-treated and control mice (C; white arrows indicate lesion sites; scale bar 500 μm), with statistical analysis (D; N = 7 per group; two-tailed unpaired Student’s *t*-test with Welch's correction; 95% *CI*[0.004962, 0.006335]

). E) IF showing Caspr expression (three fields per brain section, n = 3 brain sections per mouse, N = 5 mice per group; scale bar 10 μm). F, G) Novel object recognition test (F) and grid walking test (G) conducted at weeks 2, 4, and 9 post-stroke (N = 10 per group; one-way ANOVA followed by Tukey's post hoc test; F DIO*-Ctsd*-shRNA vs DIO-NC-shRNA: Week 2 95% *CI*[8.000, 30.60], Week 4 95% *CI*[19.77, 46.26], Week 9 95% *CI*[3.346, 21.83]; G: DIO*-Ctsd*-shRNA vs DIO-NC-shRNA:Week 9 95% *CI*[-3.434,-1.737]). H, I) Representative images of DAPI (blue), IBA1 (green), FluoroMyelin (red), and LAMP1 (purple) at week 9 post-stroke (H; white arrows indicate colocalization of FluoroMyelin with LAMP1, IBA1; scale bar 20 μm), and corresponding quantification (I; each dot represents the average of three fields per brain section, n = 3 brain sections per mouse, N = 5 mice per group; two-tailed unpaired Student’s *t*-test with Welch's correction; 95% *CI*[-46416, 65177]). Data are presented as the mean ± SEM. **p* < 0.05, ***p* < 0.01, ****p* < 0.001, *****p* < 0.0001, ns, no significance..

**Figure S11.** SLC11A1 knockdown enables lysosomes to engulf and degrade more myelin in vitro. A, B) Representative immunoblots showing SLC11A1 knockdown efficiency in primary microglia (A), and corresponding quantification (B; N = 6, six independent replicates; one-way ANOVA followed by Tukey's post hoc test; NC-shRNA vs *Slc11a1*-shRNA:95% *CI*[0.6035, 1.155]). *β*-actin served as an internal control. C, D) Representative images of DAPI (blue), eGFP (green), LAMP1 (purple), and PKH26 (red) immunostaining (C; scale bar 10 μm, zoom scale bar 5 μm), and quantification (D; three fields per plate, N = 10 plates per group; welch’s ANOVA test followed by Dunnett's T3 post hoc test; NC-shRNA+myelin vs *Slc11a1*-shRNA+myelin: 95% *CI*[-10008, -1432]). Primary microglia were infected with *Slc11a1*-shRNA or NC-shRNA lentivirus and treated with or without PKH26-labeled myelin for 3.5 h. E, F) Representative images of DAPI (blue), eGFP (green), LAMP1 (purple), and PKH26 (red) immunostaining (E; scale bar 10 μm, zoom scale bar 5 μm), and quantification (F; three fields per plate, N = 10 plates per group; welch’s ANOVA test followed by Dunnett's T3 post hoc test; NC-shRNA+myelin vs *Slc11a1*-shRNA+myelin: 95% *CI*[631.5, 4947]). Primary microglia were infected with *Slc11a1*-shRNA or NC-shRNA lentivirus and treated with or without PKH26-labeled myelin for 6 h.Data are presented as the mean ± SEM. ***p* < 0.01, *****p* < 0.0001, ns, no significance.

**Table S1.** Binding free energies and energy components predicted by MM/GBSA (kcal/mol).

| **System name** | Slc11a1/LM22B-10 | Slc11a1-Y484A/LM22B-10 |
| --- | --- | --- |
| **Δ*E*_vdw_** | -53.18±4.91 | -44.27±2.82 |
| **Δ*E*_elec_** | -4.93±4.63 | -4.39±2.34 |
| **ΔG_GB_** | 22.65±4.60 | 20.10±1.63 |
| **ΔG_SA_** | -7.02±0.44 | -6.42±0.24 |
| **ΔG_bind_** | -42.48±4.89 | -34.98±2.78 |

ΔE_vdW_: van der Waals energy.

ΔE_elec_: electrostatic energy.

ΔG_GB_: electrostatic contribution to solvation.

ΔG_SA_: non-polar contribution to solvation.

ΔG_bind_: binding free energy.

Based on the molecular dynamics trajectories, we calculated the binding free energy using the MM-GBSA method, which provides a more accurate assessment of the interaction between the small molecule and the target protein.
As shown in the table, the binding free energies of Slc11a1/LM22B-10 and Slc11a1-Y484A/LM22B-10 are –42.48 ± 4.89 and –34.98 ± 2.78 kcal/mol, respectively. Negative values indicate favorable binding affinity, with more negative values reflecting stronger binding. Clearly, our calculations show that Slc11a1/LM22B-10 exhibits stronger binding affinity. Energy decomposition reveals that the major contributions to binding come from van der Waals interactions, followed by nonpolar solvation free energy.

**Figure S12.** Key amino acid contributions to binding energy. A) SLC11A1/LM22B-10 (wild-type complex):In the wild-type complex, residues TYR504, LEU434, VAL497, and VAL481 exhibit stronger binding contributions, with maxima exceeding –3.0 kcal/mol. Notably, TYR484 itself appears among the key contributing residues, offering a greater binding energy contribution than observed after the Y484A mutation. B) SLC11A1-Y484A/LM22B-10 (mutant complex): In the mutant complex, residues such as VAL497, LEU434, and VAL481 contribute most to the binding of LM22B-10, with interaction energies ranging from –2.0 to –2.6 kcal/mol. These hydrophobic or polar residues provide a certain stabilizing effect on the ligand. However, the overall residue contributions are generally lower than in the wild type, and the loss of the critical Y484 residue likely reduces the conformational and energetic complementarity of the binding pocket.

**Figure S13.** LM22B-10 enhanced the uptake of myelin in BV2 microglia. A, B) Representative images of DAPI (blue), IBA1 (green), LAMP1 (purple), and PKH26 (red) immunostaining in BV2 microglia (A; scale bar 10 μm; zoom scale bar 2 μm), and corresponding quantification (B; three fields per plate, N = 7 plates per group;welch’s ANOVA test followed by Dunnett's T3 post hoc test; 1 μM vs 10 μM 95% *CI*[-15892, -3716],

10 μM vs 50 μM 95% *CI*[8101, 20037]). Cells were co-treated with PKH26-labeled myelin and LM22B-10 for 3.5 h. The control group received myelin without LM22B-10. Data are presented as the mean ± SEM. **p* < 0.05, ****p* < 0.001, *****p* < 0.0001, ns, no significance.

**Figure S14.** LM22B-10 is verified as a specific antagonist of SLC11A1 in vivo. A-F) Representative images of DAPI (blue), IBA1 (green), FerroOrange (red, Fe^2+^), FTH (red, Fe^3+^), FTL (red, Fe^3+^), and LAMP1 (purple) on day 7 post-stroke (A, C, E; scale bar 20 μm) in LM22B-10-treated and control groups, with corresponding quantification (B, D, F; each dot represents the average of three fields per brain section, n = 3 brain sections per mouse, N = 5 mice per group; two-tailed unpaired Student’s *t*-test with Welch's correction; FerroOrange 95% *CI*[-0.2746, -0.1755], FTH 95% *CI*[-0.2635, -0.1856], FTL 95% *CI*[-0.3611, -0.2524]

). G-N) Representative immunoblot images showing expression levels of FTH, FTL, FPN, TFR1, 4-HNE, ACSL4, and GPX4 in LM22B-10-treated and control groups (G; FTH, FTL, and 4-HNE assessed on day 7 post-stroke; ACSL4, TFR1, FPN, and GPX4 on day 4), with quantification data (H-N; FTL and TFR1, N = 4; all others, N = 5; one-way ANOVA followed by Tukey's post hoc test; Veh+L-NIO Ipsil vs LM22B-10+L-NIO Ipsil: FTH 95% *CI*[0.3257, 0.7056], FTL 95% *CI*[0.2879, 1.251], FPN 95% *CI*[-0.4020, -0.1213], TFR1 95% *CI*[0.1890, 0.6235], 4-HNE 95% CI[0.2449, 0.4407], ACSL4 95% *CI*[0.1728, 0.5640], GPX4 95% *CI*[-0.6550, -0.1824]). *β*-actin served as an internal control. Data are presented as the mean ± SEM. **p* < 0.05, ***p* < 0.01, ****p* < 0.001, *****p* < 0.0001, ns, no significance.

**Figure S15.** LM22B-10 promotes ischemic white matter recovery. A, B) Representative images of infarct volumes assessed by MBP staining (A; white arrows indicate lesion sites; scale bar 500 μm), and corresponding quantification (B; N = 7 per group; two-tailed unpaired Student’s *t*-test; 95% *CI*[-0.005412, -0.003664]). C) IF showing Caspr expression (three fields per brain section, n = 3 brain sections per mouse, N = 5 mice per group; scale bar 10 μm). D, E) Assessment of neurological deficits using the novel object recognition test (D; N = 10) and grid walking test (E; N = 10). One-way ANOVA followed by Tukey's post hoc test; D Veh+L-NIO vs LM22B-10+L-NIO: Week 2 95% *CI*[-34.91, -11.39], Week 4 95% *CI*[-35.68, -9.426], Week 9 95% *CI*[-34.13, -6.225]; E: Veh+L-NIO vs LM22B-10+L-NIO:Week 9 95% *CI*[0.6620, 2.694]. F-I) Representative images of DAPI (blue), IBA1 (green), FluoroMyelin (red), and LAMP1 (purple) at week 3 (F) and week 9 (H) post-stroke(F, H; scale bar 20 μm; white arrows indicate colocalization of FluoroMyelin with LAMP1, IBA1), and corresponding quantification (G, I; each dot represents the average of three fields per brain section, n = 3 brain sections per mouse, N = 5 mice per group; two-tailed unpaired Student’s *t*-test with Welch's correction; Week 3 95% *CI*[34977, 59625], Week 9 95% *CI*[-20123, -14031]). Data are presented as the mean ± SEM. ***p* < 0.01, ****p* < 0.001, *****p* < 0.0001, ns, no significance.

**Figure S16.** Proposed mechanism of SLC11A1 involved in the pathophysiological process of ischemic white matter injury. SLC11A1 functions as a H^+^/Fe^2+^ antiporter on the lysosomal membrane, stimulates iron transport from the cytoplasm into lysosomes, increases iron accumulation within lysosomes, destroys lysosomal lumen acidification, downgrades CTSD expression and impairs lysosomal myelin debris uptake and degradation in microglia.

**Figure S17.** The timeline of the experiments.
